# Repressed *OsMESL* expression triggers reactive oxygen species‐mediated broad‐spectrum disease resistance in rice

**DOI:** 10.1111/pbi.13566

**Published:** 2021-04-06

**Authors:** Bin Hu, Yong Zhou, Zaihui Zhou, Bo Sun, Fei Zhou, Changxi Yin, Weihua Ma, Hao Chen, Yongjun Lin

**Affiliations:** ^1^ National Key Laboratory of Crop Genetic Improvement and National Center of Plant Gene Research (Wuhan) Huazhong Agricultural University Wuhan China; ^2^ College of Bioscience and Bioengineering Jiangxi Agricultural University Nanchang China; ^3^ Wuhan Towin Biotechnology Company Limited Wuhan China

**Keywords:** JA, *Os*Trxm, ROS, rice blast, sheath blight, *Xoo*

## Abstract

A few reports have indicated that a single gene confers resistance to bacterial blight, sheath blight and rice blast. In this study, we identified a novel disease resistance mutant gene, methyl esterase‐like (*osmesl*) in rice. Mutant rice with T‐DNA insertion displayed significant resistance to bacterial blight caused by *Xanthomonas oryzae pv. oryzae* (*Xoo*), sheath blight caused by *Rhizoctonia solani* and rice blast caused by *Magnaporthe oryzae*. Additionally, CRISPR‐Cas9 knockout mutants and RNAi lines displayed resistance to these pathogens. Complementary T‐DNA mutants demonstrated a phenotype similar to the wild type (WT), thereby indicating that *osmesl* confers resistance to pathogens. Protein interaction experiments revealed that *Os*MESL affects reactive oxygen species (ROS) accumulation by interacting with thioredoxin *Os*Trxm in rice. Moreover, qRT‐PCR results showed significantly reduced mRNA levels of multiple ROS scavenging‐related genes in *osmesl* mutants. Nitroblue tetrazolium staining showed that the pathogens cause ROS accumulation, and quantitative detection revealed significantly increased levels of H_2_O_2_ in the leaves of osmesl mutants and RNAi lines after infection. The abundance of JA, a hormone associated with disease resistance, was significantly more in *osmesl* mutants than in WT plants. Overall, these results suggested that *osmesl* enhances disease resistance to *Xoo*, *R. solani* and *M*. *oryzae* by modulating the ROS balance.

## Introduction

Plants experience a wide variety of biotic and abiotic stresses during a life cycle, and diseases greatly affect crop yield and quality (Zhang and Wang, 2013). An early sign of successful cell infection in response to pathogens is a burst of reactive oxygen species (ROS) (Torres *et al.,* 2006). Previous studies have shown that elevated ROS in plants can lead to local production of programmed cell death (PCD) in leaves, thereby preventing further infection (Muhlenbock *et al.,* 2008; Ruan *et al.,* 2019). *rrsRLK* encodes a receptor‐like kinase required for ROS scavenging, and mutation of this gene results in reduced activity of enzymes involved in ROS scavenging in vivo, leading to H_2_O_2_ accumulation, which enhances defence resistance to bacterial blight (Yoo *et al.,* 2018). microRNA528 (miR528) negatively regulates rice virus defence resistance by cleaving L‐ascorbate oxidase (AO) mRNA and reduces AO‐mediated ROS accumulation (Wu *et al.,* 2017). One function of thioredoxin is to maintain ROS homeostasis in vivo. Tomato LeCITRX (Cf‐9‐interacting thioredoxin) negatively regulates plant disease defence resistance through cell death and ROS accumulation mediated by resistance protein Cf‐9 (Rivas *et al.,* 2004). Therefore, ROS is an effector that is directly or indirectly regulated by different types of genes to improve plant defence resistance when the plant is infected by pathogens.

The homologs of *OsMESL* in Arabidopsis belong to AtMES (methyl esterase) family. Previous studies have shown that *AtMES* is involved in the systemic acquired resistance (SAR) process, while its subfamily members have different functions. For example, AtMES‐1, AtMES‐2, AtMES‐7 and AtMES‐9 can hydrolyse MeSA to generate SA, and free SA acts as a long‐range SAR signal to generate a defence resistance response. Knockdown or RNA interference (RNAi)‐mediated silencing of these several genes exhibits an accumulation of MeSA in response to SAR (Vlot *et al.,* 2008), and StMES1 of potato has a similar function (Manosalva *et al.,* 2010). AtMES17 is unable to use MeSA as a substrate, but it exhibits strong hydrolysis to MeIAA in vitro (Yang *et al.,* 2008). AtMES16, which belongs to the same subfamily as AtMES17, is functionally different. AtMES16 is involved in the degradation process of chloroplasts in senescing leaves of arabidopsis and specifically catalyses the O13^4^‐demethylation of primary fluorescent chlorophyll catabolites (PFCCs) (Banala *et al.,* 2010; Christ *et al.,* 2012). However, AtMES11 which has the most similar homology with *OsMESL* and its subfamily members are yet to be reported, and they have no hydrolytic activity.

In this study, a rice *OsMESL* gene was identified. The mutants and RNAi plants showed significant defence resistance to *Xoo* and sheath blight. By contrast, the complementary plants lost defence resistance to the pathogen, and the overexpression plants showed the same phenotype with wild‐type (WT) ZH11. Protein interaction experiments showed that *Os*MESL can interact with *Os*Trxm in vivo and in vitro, thereby indicating that *Os*MESL may be involved in the ROS pathway response to pathogens. These results suggest that *Os*MESL is a key regulator of pathogen stress.

## Results

### Identification and characterization of *osmesl*

**Figure 1:**
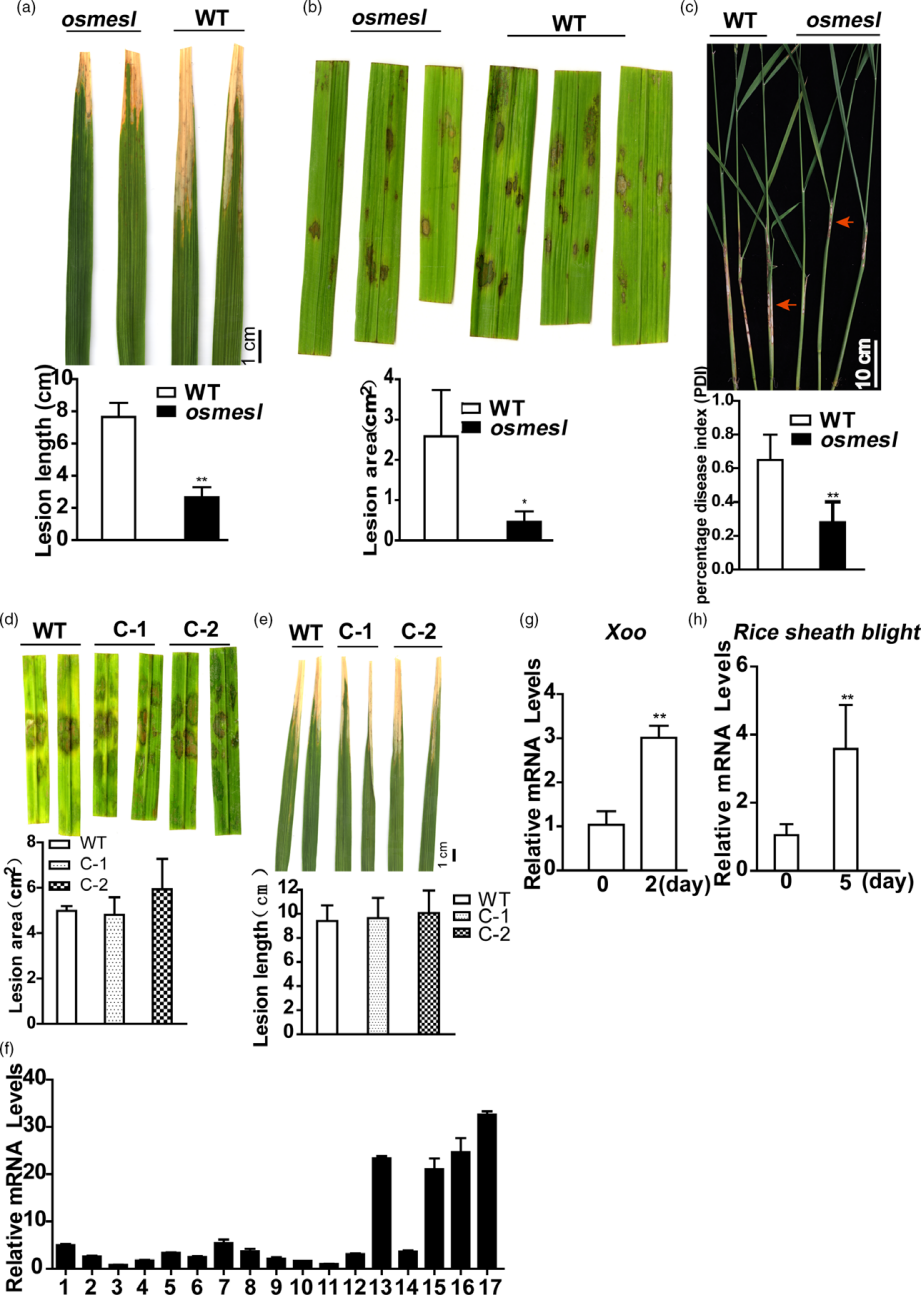
Phenotypes of mutant *osmesl* and its complementation lines, and expression pattern of *OsMESL*. (a) Phenotypes of osmesl and WT after inoculation with *Xoo*, and lesion length (cm) measurements at 10 days post‐inoculation. Values are means ± SD (*n* = 16). (b) Detached leaf phenotypes of osmesl and WT after inoculation with *R. solani*, lesion areas of sheath blight measured by photoshop. Values are means ± SD (*n* = 3). (c) Phenotypes of osmesl and WT by toothpick method after inoculation with *R. solani*. The percentage disease index. Values are means ± SD (*n* = 13). (d) Detached leaf phenotypes of WT, C‐1 and C‐2 after inoculation with *R. solani*, lesion areas of sheath blight measured by photoshop. Values are means ± SD (*n* = 3). (e) Phenotypes of WT, C‐1 and C‐2 after inoculation with *Xoo*, and lesion length (cm) measurements at 10 days post‐inoculation. Values are means ± SD (*n* = 10). (f) Detection of *OsMESL* expression in different tissues at varying developmental stages in WT. 1, Callus at 15 days after induction; 2, callus at 15 days after passage; 3, plumule at 24 h after germinating; 4, radicle at 48 h after germination; 5, seedling at three‐leaf stage; 6, aboveground part at 2 tillering stage; 7, stem at 5 days before heading; 8, stem at heading stage; 9, young panicle which length <1 mm; 10, young panicle which length was 3–5 mm; 11, young panicle which length was 10–15 mm; 12, panicled spike; 13, stamen at 1 day before flowering; 14, spikelet at 3 days after flowering; 15, flag leaf of booting stage which the panicle length was 40–50 mm; 16, flag leaf at 5 days before heading; 17, flag leaf at 14 days after flowering. (g–h) Expression of OsMESL can be induced by *Xoo* and *R. solani* in WT. Values are means ± SD (*n* = 3) (*P ≤ 0.05, **P ≤ 0.01, Student’s *t*‐test).

In this study, a T‐DNA insertion mutant was identified that demonstrated significant resistance to *Xoo* (Figure 1a) and *Rhizoctonia solani* (Figure 1b). We named this mutant *osmesl* after performing the alignment analysis with the homologous protein sequence in *Arabidopsis*. Measurement of the lesion length and areas of detached infected leaves indicated that the disease resistance conferred by *osmesl* is significantly higher than that by WT. Additionally, in vivo inoculation experiments of sheath blight conducted through the toothpick embedding method indicated enhanced resistance in *osmesl* mutants compared with WT, and the percentage disease index (PDI) of sheath blight was significantly lower in *osmesl* mutants than in WT (Figure 1c).

### Functional complementation of the *osmesl* mutant with *OsMESL* genomic DNA

We obtained the entire *OsMESL* coding region from BAC genome to construct a complementation vector for verifying the phenotype of *osmesl*. Furthermore, we genetically transformed the callus induced by *osmesl*‐homozygous positive seeds to create transgenic plants. The expression of complementary lines was detected in transgenic positive plants (Figure S1). The lines with restored expression levels, complement‐1 (C‐1) and complement‐2 (C‐2) were selected for the inoculation experiment. Results indicated that complementary lines could recover the phenotypes infected with *R. solani* (Figure 1d) and *Xoo* (Figure 1e).

### Expression pattern of *OsMESL*

Real‐time quantitative PCR results showed that *OsMESL* is constitutively expressed in all rice tissues. *OsMESL* exhibited low expression in callus, plumule, radicle, seedling, stem, young panicle and inflorescence; high expression in leaf and stamen; and the highest expression in flag leaf 14 days after heading (Figure 1f).

We analysed the expression pattern of response of *OsMESL* to pathogens in WT ZH11. The WT plants were inoculated with both pathogens, and the control was treated with water. The results indicated that *OsMESL* can be strongly induced by *Xoo* (Figure 1g) and *R. solani* (Figure 1h).

### Subcellular localization of *Os*MESL protein

The localization of *Os*MESL in chloroplasts was predicted using protein subcellular localization prediction software WoLF PSORT (https://wolfpsort.hgc.jp/). We constructed a subcellular localization vector of *Os*MESL to determine the *Os*MESL subcellular localization. We used the cauliflower mosaic virus 35S promotor (CaMV35S) to drive *Os*MESL‐mCherry and transformed rice protoplasts. Chloroplast gave white fluorescence, mCherry gave red signals and CFP gave cyan fluorescence. SCAMP was a marker located in the cell membrane. The results indicated overlapping of red fluorescence with cyan and white fluorescences. These results suggested that *Os*MESL is located in the chloroplasts and cell membrane (Figure S2).

### RNAi and CRISPR‐Cas9 of *OsMESL* in rice enhanced the resistance to pathogen infection

**Figure 2:**
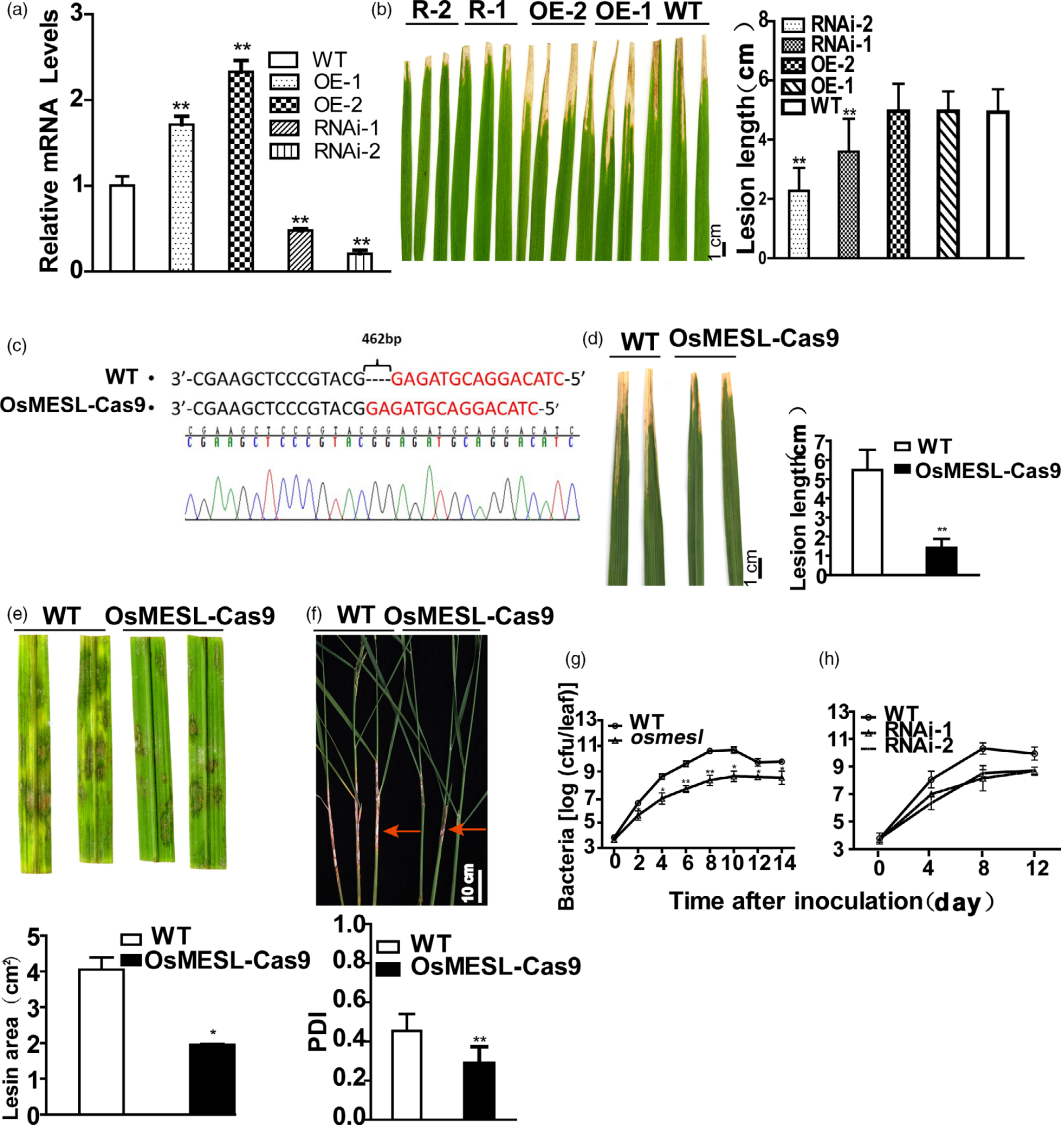
*OsMESL*‐RNAi and KO plants show resistance to *Xoo* and sheath blight, and *Xoo* growth was inhibited in *Os*MESL‐RNAi and KO plants. (a) mRNA expression levels of RNAi and OE lines. Values are means ± SD (*n* = 3). (b) Phenotypes and lesion length of RNAi‐1, RNAi‐2, OE‐1, OE‐2 and WT after inoculating with *Xoo*. Values are means ± SD (*n* = 10). (c) Sequecning of *OsMESL* KO plants for homozygous positive. (d) Phenotype and lesion length of *OsMESL* KO and WT inoculated with *Xoo*. Values are means ± SD (*n* = 13). (e) Detached leaves of *OsMESL*‐Cas9 plants inoculated with *R. solani*. Values are means ± SD (*n* = 3). (f) Phenotypes of *Os*MESL‐Cas9 plants and WT by toothpick method after inoculation with *R. solani* and the percentage disease index. Values are means ± SD (*n* = 12). (g–h) *Xoo* growth curve in WT, *osmesl* and RNAi lines. Values are means ± SD (*n* = 3) (*P ≤ 0.05, **P ≤ 0.01, Student’s *t*‐test).

We induced RNAi interference, CRISPR–Cas9 knockout and overexpression of *OsMESL* in rice to further validate the phenotypes of the T‐DNA insertion mutants. The overexpression lines, namely OE‐1 and OE‐2, RNAi lines, namely RNAi‐1 (R‐1) and RNAi‐2 (R‐2), which were single‐copy lines (Figure S3), and *Os*MESL–Cas9 KO plants were selected for further study. mRNA levels were assessed, and the mRNA level of the overexpression lines was found to be significantly higher than that of WT, whereas mRNA level of the RNAi lines was notably lower (Figure 2a). The pathogen infection test showed that the RNAi lines exhibit enhanced resistance to *Xoo*; however, no difference was observed between the OE lines and WT (Figure 2b). We obtained homozygous CRISPR–Cas9 mutants of *OsMESL* by sequencing in T_0_ transgenetic plants (Figure 2c). Inoculation with two pathogens to *Os*MESL‐cas9 KO T_1_ transgenic plants with two pathogens, the *Os*MESL–Cas9 KO mutants were also found to acquire resistance to *Xoo* (Figure 2d) and *R. solani* that not only in detached leaves (Figure 2e) but also in the living plant (Figure 2f).

To understand the growth rate of the pathogen in rice leaves, growth curves were plotted by inoculating leaves with *Xoo*. The bacterial growth rate in WT was found to be 7.95‐ to 104.27‐fold higher than in *osmesl* mutants between day 2 and day 8 (Figure 2g). However, the bacterial growth rates in WT were found to be 77.82‐ and 68.74‐fold higher than in RNAi‐1 and RNAi‐2 plants, respectively, on day 8 (Figure 2h). These results suggested that *osmesl* and RNAi plants may enhance the resistance to *Xoo* in rice by affecting the growth of the pathogen.

### H_2_O_2_ content increased and defence‐related gene expression activated in *osmesl* mutants

**Figure 3:**
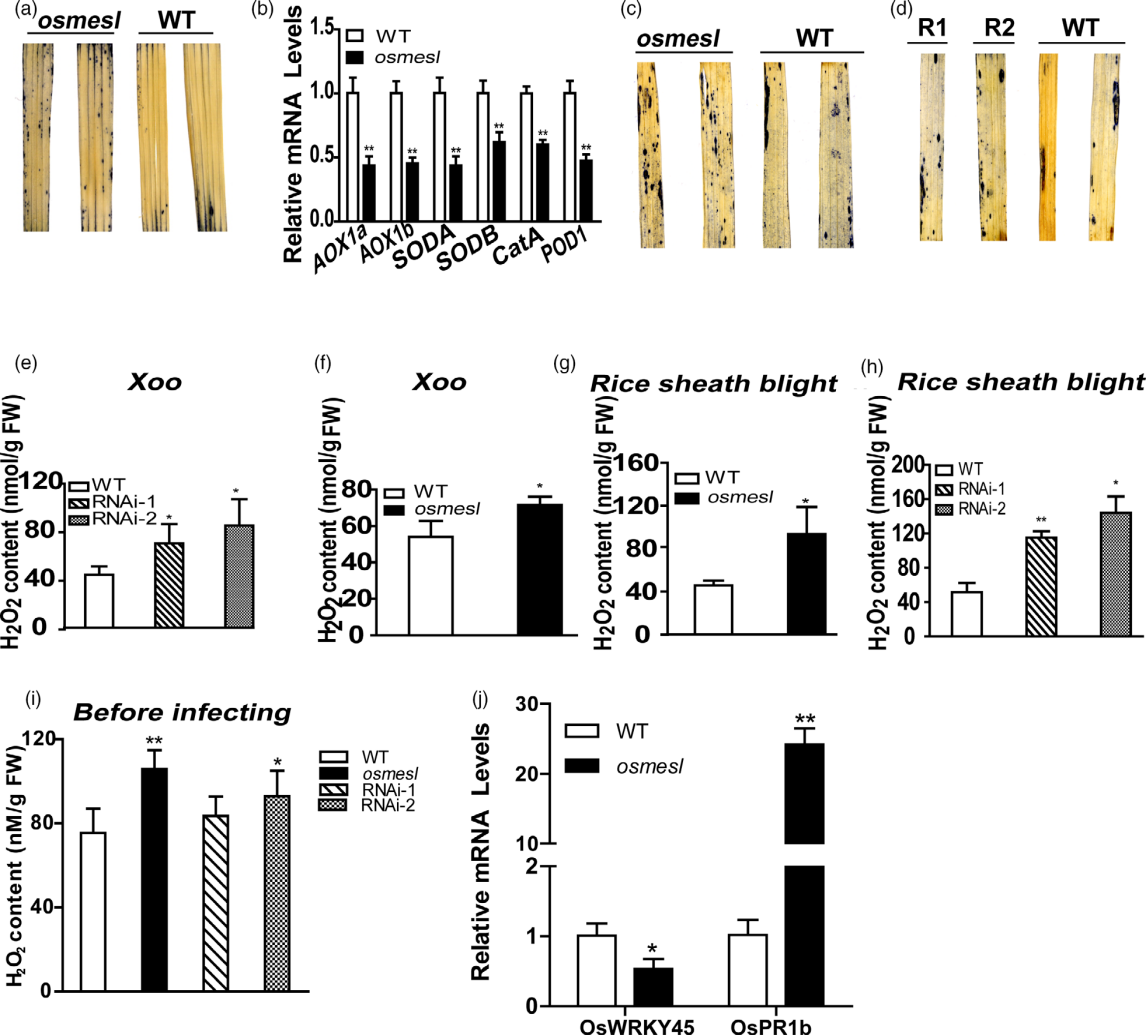
*osmesl* showed reduced ROS scavenging capacity and increased expression of genes involved in disease defence resistance. (a) NBT staining of *osmesl* and WT before inoculation with *Xoo*. (b) mRNA levels of genes involved in ROS scavenging. Values are means ± SD (*n* = 3). (c–d) NBT staining leaves of WT, *osmesl*, RNAi‐1 and RNAi‐2. (e–h) Quantitative determination of H_2_O_2_ in WT, *osmesl*, RNAi‐1 and RNAi‐2 after inoculating with *Xoo* and *R. solani,* respectively. Values are means ± SD (*n* = 3). (i) Quantitative determination of H_2_O_2_ in WT, *osmesl*, RNAi‐1 and RNAi‐2 before inoculating (*n* = 5). (j) Expression levels of genes involved in disease defense resistance. Values are means ± SD (*n* = 3) (*P ≤ 0.05, **P ≤ 0.01, Student’s *t*‐test).

Chloroplasts are the key components of the early immune response and ROS production (de Torres Zabala *et al.,* 2015). Nitroblue tetrazolium (NBT) can react with superoxide anion to form blue formazan. Hence, we performed NBT staining on leaves of *osmesl* and WT plants to verify whether chloroplast‐localized *Os*MESL is involved in the ROS pathway. The result indicated higher $O_{2}^{‐}$ content in leaves of *osmesl* plants than in those of WT plants before inoculation (Figure 3a). We further determined the mRNA expression levels of ROS scavenging genes in *osmesl* plants. The results showed that the mRNA expression levels of genes involved in ROS scavenging, namely, *AOX1a*, *AOX1b*, *SODA1*, *SODB*, *CatA* and *POD1*, in *osmesl* plants than in WT plants (Figure 3b).

Subsequently, NBT staining after inoculation with *Xoo* revealed increased ROS accumulation in *osmesl* plants and RNAi lines (Figure 3c,d). Further, we determined H_2_O_2_ content in *osmesl*, RNAi lines and WT plants after 48 h inoculation with *Xoo* or *R. solani,* respectively. The results indicated higher H_2_O_2_ content in *osmesl* plants than in WT plants (71.6 nM/g vs 54.13 nM/g FW) after *Xoo* infection, and the H_2_O_2_ content in RNAi‐1 and RNAi‐2 was found to be 70.23nM/g FW and 85.11nM/g FW, respectively, which was significantly higher than that found in WT plants (44.07 nM/g FW) (Figure 3e,f). The H_2_O_2_ content in *osmesl* plants was found to be higher than in WT plants after inoculation with *R. solani* (98.41 vs. 48.06 nM/g FW). Similarly, the H_2_O_2_ content in RNAi‐1 and RNAi‐2 was 115.05 and 144.14 nM/g FW, respectively, which was significantly higher than that found in WT plants after inoculation with *R. solani* (51.67 nM/g FW) (Figures 3g,h). We assessed whether H_2_O_2_ accumulates in the osmesl and RNAi plants before the infection. The H_2_O_2_ content in *osmesl*, RNAi‐1 and RNAi‐2 plants was 106, 83.56 and 92.99 nM/g FW, respectively, which was higher than that observed in WT plants (75.43 nM/g FW) (Figure 3i).

Further, we studied whether the expression of genes involved in disease resistance is altered in *osmesl* mutant plants. The mRNA levels of disease resistance‐related genes were determined in *osmesl* plants. The results indicated significantly increased *Os*PR1b expression in *osmesl* plants (Figure 3j). Previous studies have reported that the suppression of *Os*WRKY45‐1 expression in *japonica* rice can enhance resistance to bacterial leaf blight caused by *Xoo* and bacterial leaf streak caused by *Xoc* (Tao *et al.,* 2009). Therefore, we detected the expression of *Os* *WRKY45‐1* in *osmesl* and WT plants. The results indicated that the expression of *Os* *WRKY45‐1* is significantly decreased in *osmesl* plants compared with WT plants, after inoculation with *Xoo* (Figure 3i). These results suggested that the resistance to pathogens in *osmesl* plants may involve the ROS pathway and affect the expression of several disease resistance‐related genes.

### *Os*MESL interacts with *Os*Trxm

**Figure 4:**
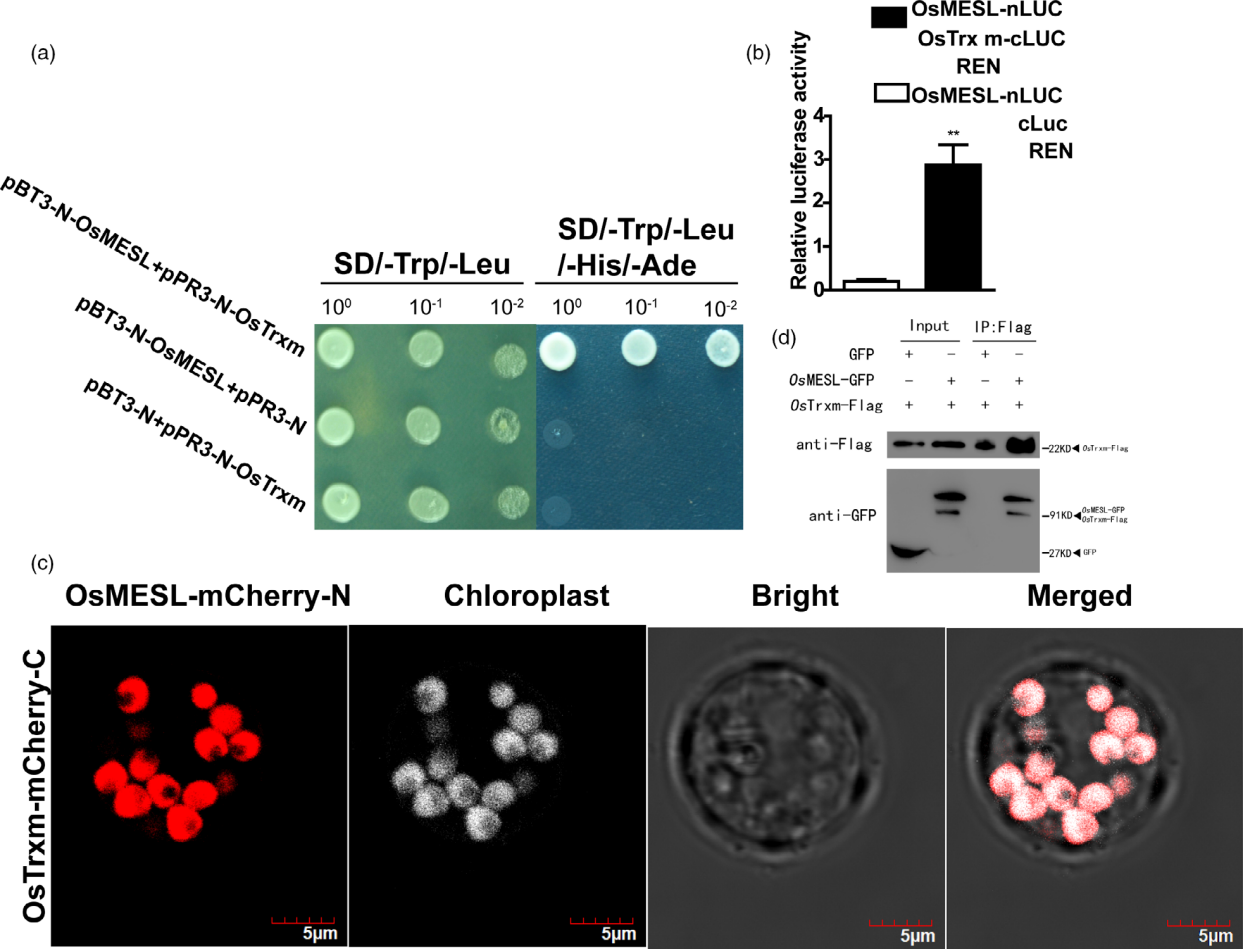
*Os*MESL interacts with *Os*Trxm in vivo and in vitro. (a) *Os*MESL interacts with *Os*Trxm in yeast NMY51. pBT3‐N‐OsMESL and vector pPR3‐N or vector pBT3‐N and pPR3‐N‐*Os*Trxm as the controls cotransformed into yeast, respectively. (b) *Os*MESL interacts with *Os*Trxm in bimolecular luciferase enzyme activity assay. Values are means ± SD (*n* = 3). (c–d) *Os*MESL interacts with *Os*Trxm in a BiFC assay. mCherry, red fluorescence. Chloroplast, white autofluorescence. Scale bars, 5 μm. (d) FLAG tagged *Os*Trxm was immunopreciptated using an anti‐FLAG antibody and co‐immunopreciptated *Os*MESL‐GFP was detected by anti‐GFP antibody. (**P ≤ 0.01, Student’s *t*‐test).

We selected a membrane‐based yeast two‐hybrid system for yeast two‐hybrid screening because of the localization of *Os*MESL in the cell membrane and chloroplast. The results indicated that *Os*MESL could interact with *Os*Trxm in yeast cells (Figure 4a). To further validate the interaction between *Os*MESL and *Os*Trxm, we performed a luciferase activity assay in rice protoplasts. The results indicated significantly higher luciferase activity in the experimental group was than in the control group, which further indicated that the two proteins could interact in vivo (Figure 4b). Additionally, we observed an interaction between *Os*MESL and *Os*Trxm in rice protoplasts through the bimolecular fluorescence complementation (BiFC) experiment by using splited mCherry (Figure 4c); the control group showed negative results. Moreover, the Co‐IP assay verified the interaction between *Os*MESL and *Os*Trxm in *Nicotiana benthamiana* (Figure 4d).

### *OsTrxm* knockout plants enhance defence to pathogens

A study reported that *Os*Trxm plays an important role in the redox regulation of chloroplast and that RNAi of *Os*Trxm increases the H_2_O_2_ content and developmental defects, such as semid‐warfism, pale‐green leaves and abnormal chloroplast structure, in rice plants (Chi *et al.,* 2008).

**Figure 5:**
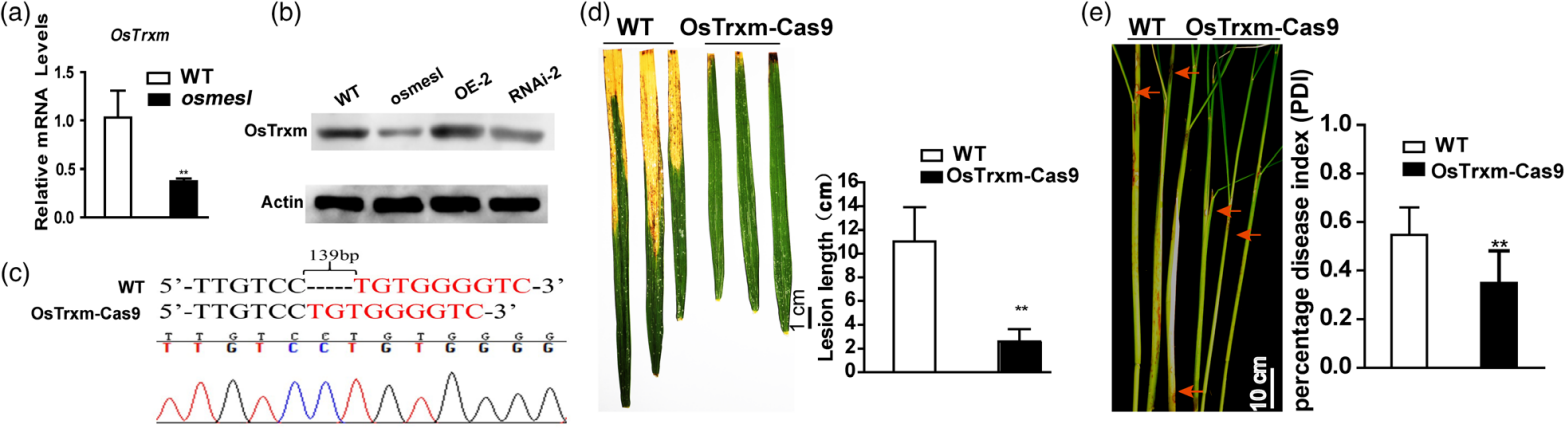
*OsTrxm*‐Cas9 plants show a defence resistance to *Xoo* and sheath blight. (a) *OsTrxm* expression level in osmesl. Values are means ± SD (*n* = 3). (b) Dection of *Os*Trxm protein level of osmesl, OE‐2, RNAi‐2 by Western blot. (c) Sequencing of *OsTrxm* KO plants for homozygous positive. (d) Phenotype of *OsTrxm*‐Cas9 plants after inoculating with *Xoo*, and lesion length (cm) measurements at 10 days post‐inoculation. Values are means ± SD (*n* = 13). (e) Phenotype of *OsTrxm*‐Cas9 plants after inoculating with *R. solani*. Values are means ± SD (*n* = 13) (**P ≤ 0.01, Student’s *t*‐test).

We first examined the mRNA expression level of *OsTrxm* in *osmesl* mutants to understand whether the mRNA expression level of *Os*Trxm is altered. The results showed a significantly lower expression of *OsTrxm* in *osmesl* mutants than in WT plants (Figure 5a). Furthermore, we measured the protein content of *Os*Trxm in *osmesl* mutants, OE and RNAi lines by using the Western blot assay. We observed that the protein content of *Os*Trxm in *osmesl* and RNAi‐2 plants is lower than in WT. Therefore, we constructed *OsTrxm*‐Cas9 knockout and *35S::Trxm* plants to determine the phenotypes. A DNA sequence of 139bp length, which included parts of the sequence of the first exon and first intron, was knocked out (Figure 5c). Inoculation with pathogens *Xoo* and *R. solani* revealed increased resistance to *Xoo* (Figure 5d) and sheath blight (Figure 5e) in *OsTrxm*–Cas9 homozygous mutant plants, compared with the control. However, the *Os*Trxm‐OE lines displayed no difference in resistance to both pathogens (Figure S4). These results illustrated that *Os*Trxm may be crucial for the involvement of *osmesl* in ROS‐mediated disease resistance pathways.

### JA signalling pathway activation in *osmesl* mutant

**Figure 6:**
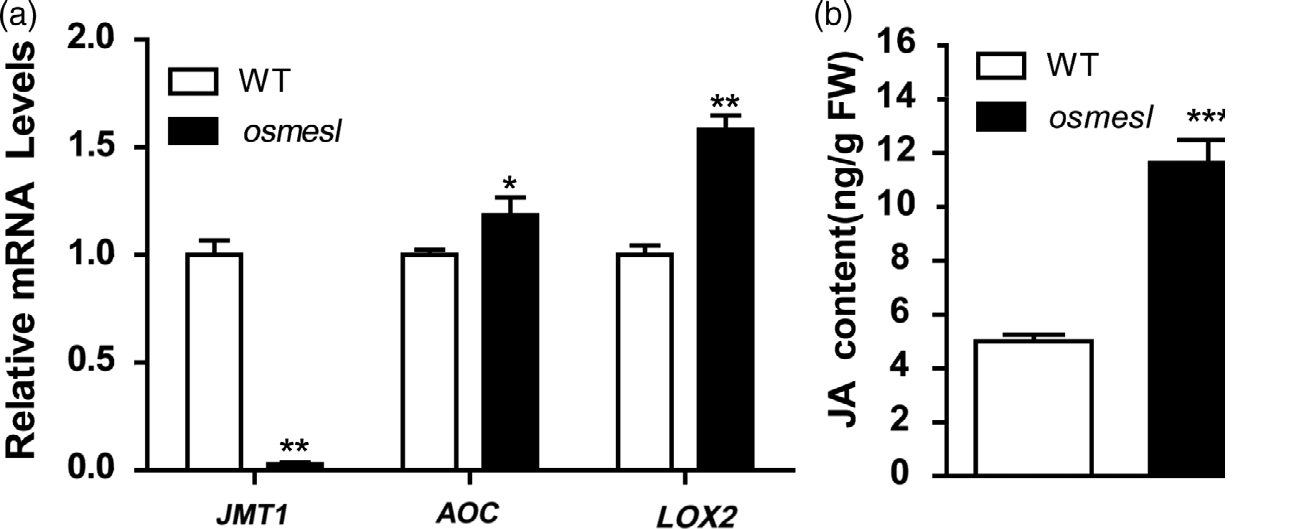
JA quantitative determination and related gene expression level. (a) JA signaling pathway‐related gene expression level. Values are means ± SD (*n* = 3). (b) JA content in *osmesl* and WT. Values are means ± SD (*n* = 3) (*P ≤ 0.05, **P ≤ 0.01, ***P ≤ 0.001, Studment’s *t*‐test).

The phytohormone JA has an important role in regulating immune responses (Galis *et al.,* 2009; Yan and Xie, 2015; Yu *et al.,* 2020). Studies have shown that the overexpression of a *Os*Trxm interacting protein BAS1, a 2‐Cys peroxiredoxins, increases the plant tolerance to alkyl hydroperoxides. BAS1 could control the synthesis of JA by reducing 13‐hydroperoxy linolenic acid (Baier and Dietz, 1997; Zhang *et al.,* 2011). Thus, we assessed whether the decreased *Os*Trxm expression affects the JA pathway in *osmesl* mutants. First, we assessed the expression of certain genes in the JA synthesis and signalling pathway in *osmesl* mutants. The expression of *AOC* and *LOX2*, the key genes involved in the JA synthesis pathway, was found to be significantly higher, whereas that of *JMT1*, an MeJA synthesis gene, was found to be significantly lower in *osmesl* mutants than in WT plants (Figure 6a). These results indicated that the JA content may be higher in *osmesl* mutants than in WT plants. Subsequently, we quantitatively determined the JA content of *osmesl* mutants and WT plants at the seedling stage. The JA content in *osmesl* mutants was found to be higher than in WT plants (11.63 vs 5.01 ng/g FW) (Figure 6b). Thus, the results indicate that the resistance to pathogens conferred by *osmesl* may involve the JA signalling pathway.

### Activation of defence response and inhibition of ROS scavenging‐related gene expression revealed through RNA‐Seq

To further unravel the function of *OsMESL* in rice, heading stage leaves in WT and *osmesl* plants were used for the transcriptome analysis. A total of 3602 differentially expressed genes (DEGs) were identified (*P* value < 0.05). Of these genes, 2052 were up‐regulated and 1550 were down‐regulated in *osmesl* plants. GO terms were significantly involved in metabolic process, response to stress, catalytic activity, response to biotic stimulus and response to abiotic stimulus (Figure S5). A set of DEGs associated with defence response [namely *WRKY24* (Yokotani *et al.,* 2018), *WRKY77* (Lan *et al.,* 2013), *Pi9* (Qu *et al.,* 2006), *WRKY76* (Liang *et al.,* 2017), *MADS26* (Khong *et al.,* 2015), *PR1a* (Agrawala, 2001), *JIPR10* (Jwa *et al.,* 2001), *RSPR10* (Hashimoto *et al.,* 2004) and *DR8* (Wang *et al.,* 2006)] and ROS scavenging [namely *AOX1a* (alternative oxidase), *POD1* (peroxidase), *CATB* (catalase), *MT2b* (metallothionein) (Wong *et al.,* 2004), *APX1* (ascorbate peroxidases), *APX3*, *APX8* (Singh and Shah, 2014)] was found to be significantly down‐regulated in *osmesl* plants (Table S2). Genes involved in the JA synthesis pathway, namely *GH3.5* (jasmonic acid amino acid synthase), *OPR1* and *OPR7* (12‐oxo‐phytodienoate reductase gene) (Sobajima *et al.,* 2007; Tani *et al.,* 2008), *LOX1*, *LOX2*, *LOX5*, *LOX6*, *LOX8* and *LOX9* (lipoxygenase gene) (Zhang *et al.,* 2018), were found to be up‐regulated in the transcriptome data. Meanwhile, the mRNA level of the *Os*Trxm interaction protein, BAS1, was found to be significantly lower in *osmesl* mutants than in WT in the transcriptome data, which indicated that the expression of BAS1 is repressed in mutants. The expression of the lipid hydroperoxide lyase gene (*HPL3*), which negatively regulates JA synthesis, was found to be decreased in the transcriptome data. *HPL3* negatively regulates JA synthesis mainly by hydrolysing hydroperoxide linolenic acid to produce green leaf volatiles (Liu *et al.,* 2012b; Tong *et al.,* 2012). Thus, the results suggest that the mutation of *OsMESL* might affect the expression of defence response‐ and ROS scavenging‐related genes to some extent.

## Discussion

In this study, resistance to *Xoo* and sheath blight was observed in the disease‐resistant mutant *osmesl*, RNAi and *OsMESL* knockout plants. Complementation of *osmesl* can restore the phenotype sensitive to pathogens, which suggests that the phenotype of *osmesl* plants is indeed because of *OsMESL* mutation.

### Accumulation of ROS in *osmesl* mutants confers broad‐spectrum disease resistance

Plants can adopt various strategies to resist pathogen infection. In maize, ZmFBL41 gene knockout confers resistance to banded leaf and sheath blight by accumulating lignin (Li *et al.,* 2019a). *Os*Xa13 protein interacts with two copper transporters, COPT1 and COPT5, and co‐ordinates the distribution of copper, which is toxic to *Xoo* in rice (Yuan *et al.,* 2010). Similarly, *Os*XA3/XA26 interacts with a triosephosphate isomerase (TPI), *Os*TPI1.1, and modulates ROS production through the glycolysis pathway (Liu *et al.,* 2018). Chloroplasts, one of the major sources of ROS, are the main sites that are involved in $O_{2}^{‐}$ production. $O_{2}^{‐}$ is unstable and its subsequent catalys is by dismutases generates H_2_O_2_, which in turn is hydrolysed by peroxidases or catalases (CAT) to produce H_2_O and O_2_ (Krieger‐Liszkay, 2005). Recent studies have revealed that ROS can also act as a signalling molecule that regulates various stresses, such as plant growth and development, hypersensitive response and pathogen‐induced SAR (Baxter *et al.,* 2014; Zhang *et al.,* 2014).

ROS plays an important role in plant–pathogen interactions (Heller and Tudzynski, 2011). ROS‐related plant defence responses include direct killing of pathogens that activates PCD and provides a suitable redox environment for the initiation of immune responses (Vellosillo *et al.,* 2010). Moreover, ROS offer wide possibilities for broad‐spectrum disease resistance (Li *et al.,* 2019b; Li *et al.,* 2017). Protein interaction experiments have demonstrated that *Os*MESL can interact with *Os*Trxm, which is localized in chloroplasts. Previous studies have shown that thioredoxin (TRX), an antioxidant modulator, can improve the tolerance of TRX‐deficient yeast to oxidative stress (Issakidis‐Bourguet *et al.,* 2001). TRX localized in chloroplast regulates the activity and functions of various enzymes present in chloroplasts. A commonly studied TRX target protein is NADPH–malate dehydrogenase (NADPH–MDH), which is involved in exporting the reducing energy from chloroplasts to the cytosol in the form of malate with complete inactivation of the oxidized state of NADP–MDH, and its activity is strictly dependent on TRX (Arner and Holmgren, 2000; Lemaire *et al.,* 2007; Motohashi *et al.,* 2001). XopI, an F‐box effector of a *Xoo* strain BAI3, acts as an adapter to form a ternary complex, which regulates the immune response in rice through proteasomal degradation of *Os*Trxh2 (Ji *et al.,* 2020). *OsTrxm* RNAi plants exhibit certain phenotypes, such as increased H_2_O_2_ levels, abnormal chloroplast structure and disrupted ROS scavenging (Chi *et al.,* 2008). In the present study, *OsTrxm* expression was significantly reduced in *osmesl* mutants, and the mutants exhibited ROS accumulation before and after infection by pathogens. Given that previous studies did not address the relationship between *OsTrxm* and disease resistance, we assessed whether the resistance to pathogens is because of increased ROS levels in *osmesl* mutants and the decreased expression level of *OsTrxm*. H_2_O_2_, JA and defence‐related genes *PR1b* and *LOX2* were increased in the *Os*Trxm knockout plants (Figures S6A–D). Further, *OsTrxm* knockout plants were inoculated with *Xoo* or *R. solani*. In *OsTrxm* knockout plants, resistance to pathogens was found to be enhanced. Furthermore, we explored whether *osmesl* mutants are resistant to rice blast. We induced rice blast in *osmesl* mutants and found that *osmesl* mutants are significantly resistant to rice blast (Figure S7A,B). Similarly, RNAi‐1 and RNAi‐2 also displayed resistance to rice blast (Figures S7C,D). These results suggest that *Os*MESL is involved in broad‐spectrum resistance to pathogens in rice.

### Resistance to pathogens by *osmesl* involves the JA signalling pathway

JA, as a hormone for plant defence responses, plays an important role in disease defence resistance. The relationship between JA and ROS has not been well studied. Previous studies have reported that rice WRKY13 activates the glutathione/glutaredoxin system, which maintains the ROS balance, but inhibits JA biosynthesis in pathogen‐induced defence responses (Qiu *et al.,* 2007). The exogenous application of methyl jasmonate to castor bean leaves has been found to induce ROS accumulation and decrease the activities of ROS scavenging enzymes, namely superoxide dismutase, CAT and guaiacol peroxidase. Recent studies have found that JA production can be induced by H_2_O_2_ treatment in Arabidopsis (Camejo *et al.,* 2016; Hieno *et al.,* 2019; Singh *et al.,* 2016). In tomato leaves, wounding or JA treatment has been found to significant increase the activity of plasma membrane NADPH oxidase and ROS accumulation; however, the accumulation of ROS was found to be blocked by pre‐treatment with plasma membrane NADPH oxidase inhibitor or H_2_O_2_ scavengers (Li *et al.,* 2002; Wasternack *et al.,* 2006). These studies illustrated a certain relationship between JA and ROS. In the present study, the key genes, *AOC* and *LOX2*, of the JA synthesis pathway were significantly up‐regulated in *osmesl* mutants. However, the expression of *JMT1*, a MeJA synthase gene, was significantly decreased. These results indicated that the JA signalling pathway is activated in *osmesl* mutants. Quantitative determination of JA revealed that the JA content in *osmesl* mutants is twice of that in WT plants at the seedling stage. We speculated that *OsMESL* regulates ROS scavenging and involves activation of the JA signalling pathway by inducing ROS imbalance.

Previous studies have reported that BAS1, an interaction protein of *Os*Trxm, has a reductase effect on H_2_O_2_ and alkyl hydroperoxides. Additionally, JA synthesis can be regulated by reducing 13‐hydroperoxide linolenic acid. JA is synthesized from linolenic acid, which undergoes a series of oxidation reactions, followed by reduction reactions, to finally produce JA. Our transcriptome data indicated a decreased BAS1 expression in the mutant. We speculate that the decreased BAS1 expression could decrease the reducing potential of linolenic acid, further facilitating the oxidative response to linolenic acid and promoting the initiation of JA synthesis. Meanwhile, the key genes of *Os*AOC and *Os*LOX2 in JA synthesis pathway up‐regulated in *osmesl* mutants. In *osmesl* mutants, ROS scavenging is impaired and the reduction force in vivo is low. Previous studies have reported that the mRNA level of BAS1 decreases with increasing concentrations of glutathione and ascorbic acid; however, two of the most important antioxidants in vivo and oxidative stressors, such as methyl viologen and light, slightly induce its expression (Dietz, 1994). However, in *osmesl* mutants, the expression of *BAS1* was instead significantly reduced in the environment with low reducing power, which seems to be contradictory to the function of BAS1. A possible reason for decreased *BAS1* expression could be the impaired *Os*Trxm. In fact, the expression of *BAS1* was down‐regulated in *Os*Trxm–Cas9 plants (Figure S8). Similarly, the RNA‐Seq data indicated that *HPL3*, which could hydrolyse the hydroperoxy polyunsaturated fatty acid, down‐regulated in *osmesl* mutants; hydroperoxy polyunsaturated fatty acid is the substrate of JA synthesis. *Os*Trxm was down‐regulated after MeJA induction in *Zea mays* (Zhang *et al.,* 2015). Considering that *Os*MESL has an N‐myristoylation site at the N‐terminal, we speculated that *Os*MESL plays an important role in the stability of *Os*Trxm. The western blot analysis showed that the reduced *Os*MESL gene expression decreases the protein content of *Os*Trxm. These results suggest that *osmesl* confers pathogen resistance through the ROS and JA pathways by affecting *Os*Trxm protein stability.

**Figure 7:**
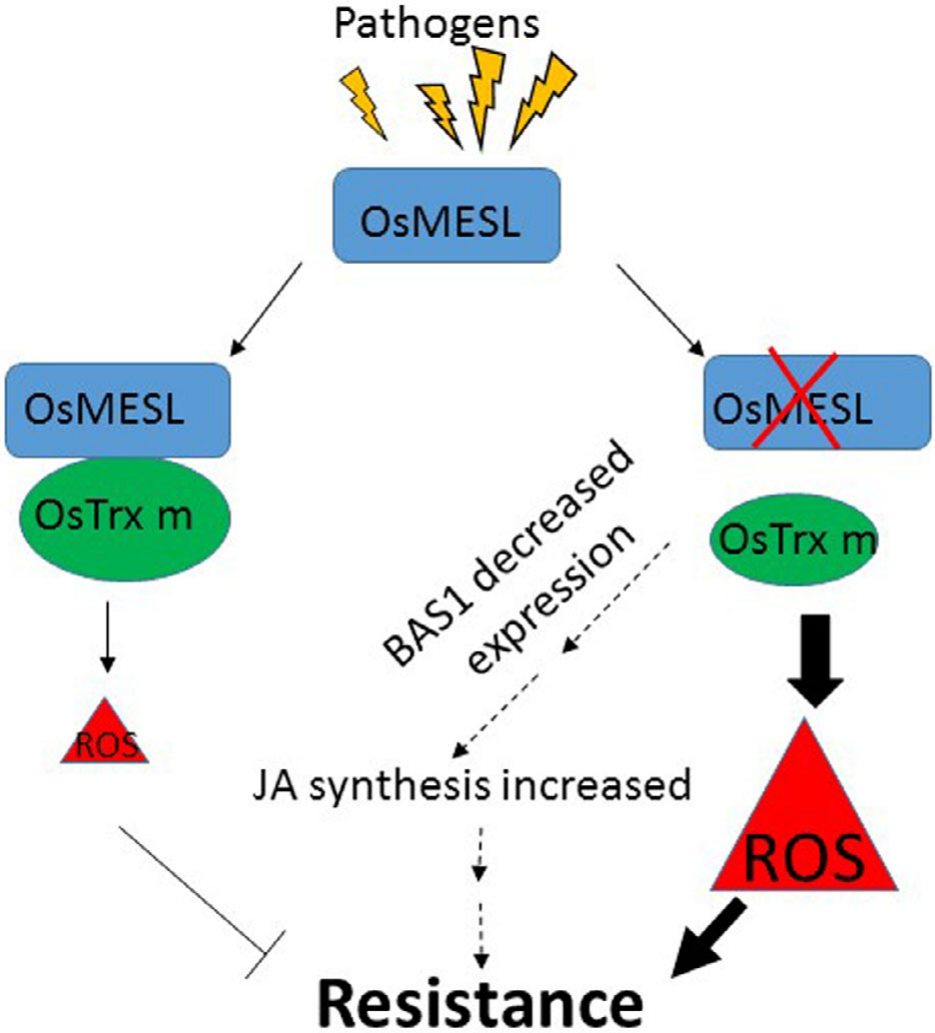
Model for the role of *Os*MESL in the resistance to *Xoo* and *R. solani*. *Os*MESL assists *Os*Trxm in ROS scavenging. When *Os*MESL expression is supressed, the *Os*Trxm ROS scavenging capacity is attenuated, and resulting in ROS accumulation. In addition, JA synthesis is increased due to *BAS*1 decreased expression, and some resistance gene induced expression in *osmesl*. Finally, these results lead to the resistance to the pathogens.

In summary, we proposed a working model for the role of *OsMESL* (Figure 7). *Os*MESL somehow assists *Os*Trxm in ROS scavenging. When *Os*MESL expression is reduced, the ROS scavenging capacity of *Os*Trxm is attenuated, which results in ROS accumulation. In addition, the increase in JA synthesis could be attributed to the decreased expression of *BAS1*, and the expression of some resistance gene is induced in *osmes* mutantsl. Finally, repressed *OsMESL* confers resistance to different pathogens.

## Materials and methods

### Plant material and growth condition

In this study, the japonica rice (*Oryza sativa*) cultivar (Zhonghua 11, ZH11) was used as the WT control, and transformation was performed on this cultivar. The *osmesl* T‐DNA insertion mutant was procured from Kyung Hee University, Republic of Korea. The mutant family number was 3A‐03499. The primers used for the genotype detection were MESLT‐F, MESLT‐R and 2715L2. The field was sown and transplantation was performed after approximately 1 month, with 10 plants per row for a total of 3 rows. Agronomic character investigation and sample collection were conducted in the field under natural conditions during summer in Wuhan.

Samples from different parts of various tissues were obtained for analysing the expression pattern of *Os*MESL in WT rice. The samples were as follows: (1) callus 15 days after induction, (2) callus 15 days after passage, (3) plumule 24 h after germination, (4) radicle 48 h after germination, (5) seedling at the three‐leaf stage, (6) part above the ground at 2 tillering stage, (7) stem 5 days before heading, (8); stem at the heading stage, (9) young panicle with length of <1 mm, (10) young panicle with length of 3–5 mm, (11) young panicle with length of 10–15 mm, (12) panicled spike, (13) stamen 1 day before flowering, (14) spikelet 3 days after flowering, (15) flag leaf of booting stage with panicle length 40–50 mm, (16) flag leaf 5 days before heading and (17) flag leaf 14 days after flowering.

### Vector construction and genetic transformation

The total RNA from WT ZH11 leaves was extracted and reverse transcribed to form the cDNA. The *OsMESL* (LOC_*Os*07g41230) gene was amplified from full‐length cDNA and constructed onto the pU1301 vector with cauliflower mosaic virus (CaMV) 35S RNA as the promoter. The RNAi vector amplified a 227‐bp fragment from the cDNA sequence of *OsMESL* and constructed in the pDS1301 vector. The BAC clone containing the *OsMESL* gene was selected from the Nipponbare BAC library in our laboratory, and the clone number was A0053G01. The target fragment was ligated into the final vector pC2301 by using a restriction enzyme to construct the complementation vector. *OsMESL*–Cas9 and *OsTrxm*–Cas9 were used to drive the expression of gRNA by using U3 and U6a promoters through dual‐target knockdown. WT ZH11 was transformed using *Agrobacterium*‐mediated genetic transformation (Lin and Zhang, 2005). Table S1 lists all the primers used in the experiment.

### RNA extraction and qRT‐PCR analysis

The tissue samples, such as leaves, were immediately frozen in liquid nitrogen after sampling from the field. The total RNA was extracted with TRIzol reagent (TransGen Biotech, Beijing, China) as per the manufacturer’s instructions. Approximately 2 µg of total RNA was reverse transcribed, and the first strand of cDNA was obtained by reverse transcription by using Superscript III reverse transcriptase kit (Invitrogen, California, USA) as per manufacturer’s instructions. qRT‐PCR experiments were performed using FastStar Universal SYBR Green Master (ROX) (Roche, Basel, Switzerland) kits on an ABI 7500 system (Thermo Fisher Scientific, California, USA). The relative expression was calculated. The rice actin gene was used as an internal reference (Livak and Schmittgen, 2001). The primers used in the experiment are listed in Table S1.

### Subcellular localization assays

The full‐length cDNA of *Os*MESL was constructed into the pM999‐mCherry vector through homologous recombination. The *Os*MES11–pM999‐mCherry plasmid was transformed into rice protoplasts for transient expression. Images were captured using a confocal fluorescence microscope (Olympus FV1200). Cyan fluorescence from the CFP channel, red fluorescence from the mCherry channel and chloroplast fluorescence were observed at the excitation wavelengths of 405, 559 and 488 nm, respectively, and the fluorescence acquisition wavelengths of 460–500, 570–618 and 655–755 nm, respectively.

### Protein interaction analysis

The membrane system yeast two‐hybrid library constructed in our laboratory was used to screen the library. The entire rice plant at the seedling stage was used for constructing the cDNA library. The ligation vector was pPR3‐N, and *OsMESL* constructed on the pBT3‐N vector was used to screen the library. *Os*Trxm was constructed in pBT3‐N for interaction validation in yeast. All the restriction sites used in the study were SfiI. PEG4000 was used to transform the yeast strain *NMY51*; the yeast culture temperature was 30 °C, and the 3‐AT screening concentration was 25 mm.

The BiFC assay was performed in rice protoplast. Rice protoplast isolation and transformation were performed according to a previously described method (Tang *et al.,* 2012; Xie *et al.,* 2015). The rice *OsMESL* gene was cloned into the 1300s‐mCherry‐N vector, and *OsTrxm* was cloned into the 1300s‐mCherry‐C vector (Fan *et al.,* 2008). Rice protoplasts were isolated from 12‐day‐old seedlings of ZH11 (*Oryza sativa* spp. japonica). First, the rice protoplasts were isolated by digesting the rice sheath strips in digestion solution (10 mm MES, pH 5.7; 0.6 m mannitol; 1 mm CaCl_2_; 5 mm beta‐mercaptoethanol; 0.1% BSA; 0.3% cellulase RS; and 0.75% macerozume R10) for 4 h with gentle agitation. Further, the protoplasts were incubated in W5 solution for 30 min, precipitated through centrifugation at 100 ×g for 8 min and finally resuspended in MMG solution. For transformation, 7 μg of each plasmid was joined together and gently mixed with 100 μL of protoplasts and 110 μL of PEG–CaCl_2_ solution, and the mixture was further incubated at room temperature for 15 min in dark. W5 solution (twice the volume of reaction mixture) was added to stop the transformation. The transformed protoplasts were collected through centrifugation and further resuspended in WI solution. The transformed protoplasts were incubated for 12 h in dark. Finally, the protoplasts were collected through centrifugation at 100 ×g for 8 min and immediately subjected to the fluorescence analysis (Olympus FV1200).

The protein interaction of luciferase activity recovery method was performed in rice protoplasts. Plasmids with N‐ and C‐terminal LUC fusion genes were cotransformed with *35S:REN* in the ratio 10:10:1, with the latter as the internal control. The REN and LUC activities were detected using REN and LUC assay substrates (Promega, Madison, WI, USA), respectively, after 20‐h incubation. Relative activity of the LUC reporter was expressed as the ratio of LUC to GUS (Wang *et al.,* 2015).

Co‐immunoprecipitation assay (Co‐IP) was performed in the *Nicotiana benthamiana* system. The *OsMESL* coding region was constructed in fusion with a C‐terminal GFP tag in pC1300S‐GFP, and OsTrxm was constructed in fusion with a C‐terminal FLAG tag in PU2301‐Flag. The total protein was extracted from the leaves of *N. benthamiana* for the Co‐IP assay. The Co‐IP samples were subjected to western blot by using anti‐GFP (1:10,000 dilution; ab290, Abcam, Cambridge, UK) and anti‐FLAG (1:10,000 dilution; F3165, Sigma‐Aldrich, St. Louis, MO, USA) antibodies.

Overall, 50 μg of each sample was used for western blot. Actin (1:2000 dilution, #A01050, Abbkine) was used as the control. Antibodies were prepared using 73 C‐terminal amino acids of OsTrxm as the antigen. Anti‐OsTrxm was used in 1:1000 dilution for western blot.

### Pathogen inoculation

The blight species was Philippine species PXO99 (race 6). Flag leaves at the booting stage of rice were selected for the inoculation experiment by using the leaf‐clipping method (Zhou *et al.,* 2002). Disease was scored by measuring the lesion length 10 days after inoculation. Bacterial growth in rice leaves was measured by counting the colony‐forming units (Sun *et al.,* 2004).

The pathogen of sheath blight was *RH‐9* virulent strain. Two inoculation methods were used in this study. Flag leaves with the same growth status at the booting stage in field were selected in vitro. Sheath blight fungi were placed in the middle of the detached leaves, moisturized, cultured at 28 °C for 48 h, and further photographed. The relative lesion area was measured using Photoshop. In the fields, toothpick embedding method was used to inoculate *R. solani*. Disease severity was measured as the relative lesion height and expressed as the percentage disease index (Molla *et al.,* 2013).

### NBT staining and quantitative determination of H_2_O_2_

NBT (Sigma‐Aldrich, St. Louis, MO, USA) powder was dissolved in sterile ddH_2_O. The leaves, which were grafted with the disease for approximately 7 days, were stained in dark at 28 °C. Subsequently, the leaves were destained with 95% ethanol. Finally, 40% glycerol was used for rehydration for photography.

Flag leaves after 48 h of inoculation with *Xoo* or *R*. *solani* were subjected to quantitative determination of H_2_O_2_ in the tillering stage. The part of leaves approximately 10 cm below the incision was obtained and immediately placed into liquid nitrogen for H_2_O_2_ determination. Overall, 3 biological replicates per sample were used. The whole flag leaf before infection was subjected to quantitative determination of H_2_O_2_ in the late tillering stage. Overall, 5 biological replicates were used per sample. The procedures were performed using Amplex Red Hydrogen Peroxide/Peroxidase Assay Kit (Thermo Fisher Scientific, CAS: A22188) according to the manufacturer's instructions.

### Determination of endogenous JA in rice

The parts of *osmesl* and WT ZH11 plant seedlings above the ground at the three‐leaf stage were immediately frozen in liquid nitrogen (four biological replicates per sample). The samples from *Os*Trxm‐Cas9 plants were collected in the late stage of rice maturation. Hormone extraction assays were performed using a method described previously (Liu *et al.,* 2012a).

### Transcriptome sequencing and data analysis

RNA samples from WT and *osmesl* plants at the heading stage were used for transcriptome sequencing, and each sample was pooled for total RNA isolation (6 biological replicates). Transcriptome sequencing was performed in CapitalBio Technology Corporation by using the Illumina HiSeq X ten system according to the standard procedure. Raw reads were subjected to quality check by using ‘FastP’ (Chen *et al.,* 2018). Sample‐wise high‐quality reads were aligned to the rice reference genome (http://rice.plantbiology.msu.edu/) by using ‘HISAT2’ (Kim *et al.,* 2015). Aligned reads were subsequently used to create a reference annotation‐based transcript assembly to identify uniquely aligned reads in each sample. The results were subjected to ‘featureCounts v1.5.0’ (Liao *et al.,* 2014) to obtain the read count of all samples. Differences in the gene expression were identified using ‘DESeq2 1.2.5R’ (Love *et al.,* 2014). The thresholds for P value and FDR (adjusted *P* value or *Q*‐value) were set to 0.05 for identifying significant differences in the expression. Gene ontology was derived from the msu7 rice genome database.

## Accession numbers

The sequence data from this article can be found in the RGAP database (http://rice.plantbiology.msu.edu) under the following accession numbers: *OsMESL*, LOC_*Os*07g41230; *OsTrxm*, LOC_*Os*12g08730; *SODB*, LOC_*Os*06g05110; *SODA1*, LOC_*Os*05g25850; *CatA*, LOC_*Os*02g02400; *POD1*, LOC_*Os*07g02440; *AOX1a*, LOC_*Os*04g51150; *AOX1b*, LOC_*Os*04g51160; *PR1a*, LOC_*Os*07g03710; *PR1b*, LOC_*Os*01g28450; *PR10*, *WRKY45‐1*, LOC_*Os*05g25770; *JMT1*, LOC_*Os*06g20920; *AOC*, LOC_*Os*03g32314; *LOX2*, LOC_*Os*03g08220; *BAS1*, LOC_*Os*02g33450 and *Actin*, LOC_*Os*03g50885.

## CONFLICT OF INTERESTS

The authors have no conflicts of interest to declare.

## Author contributions

Y.L. and B.H. conceived and designed the experiments. B.H. performed the experiments and wrote the manuscript. Y.L. edited and revised the manuscript. Y.Z. provided assistance with the writing of the manuscript, and Z.Z. provided assistance with the luciferase activity assay. All other authors participated in the discussion of the results and commented on the manuscript.

## Supporting information

**Figure S1** The expression level of complementary lines.

**Figure S2** Subcellular localization of *Os*MESL protein.

**Figure S3** Detectiion of copy numbers in *OsMESL*‐RNAi lines, *OsMESL*‐OE lines and *osmesl*complementation lines

**Figure S4** Phenotypes of *OsTrxm* OE lines inoculated with *Xoo* and *R. solani*.

**Figure S5** GO analysis of DEGs in *osmesl* and WT.

**Figure S6** Detection of disease resistance‐related indicators.

**Figure S7***osmesl* and RNAi lines showed resistance to rice blast.

**Figure S8** Expression level of *OsBAS1* in OsTrxm‐Cas9 mutant.

**Table S1** Primers used in this study.

**Table S2** Differentially expressed of defence response‐ and ROS scavenging‐related genes in *osmesl* plants.
