# Supplementary figures and images for: Repressed OsMESL expression triggers reactive oxygen species‐mediated broad‐spectrum disease resistance in rice
Source: Plant Biotechnol J. 2021 Apr 6;19(8):1511–22. doi: 10.1111/pbi.13566 (PMC8384603; doi:10.1111/pbi.13566)

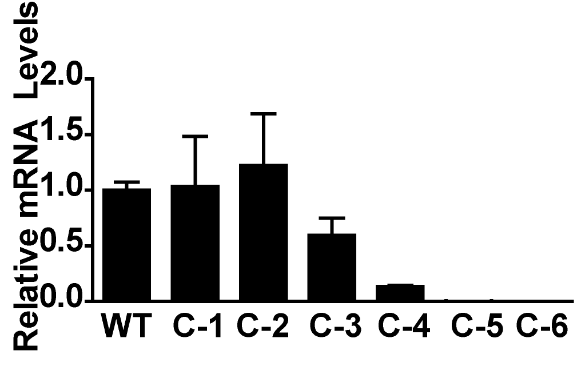


**Supplemental Figure S1.** The expression level of complementary lines.

Supplement: Supplementary file 1 — Figure S1 The expression level of complementary lines. [file PBI-19-1511-s005.docx]

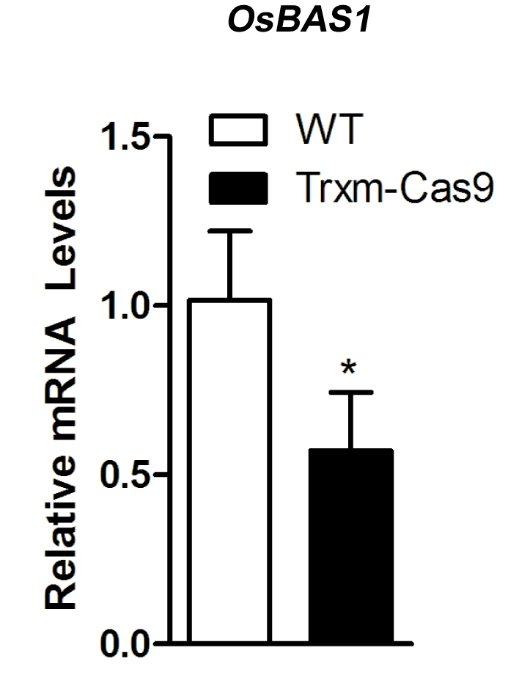


**Supplemental Figure S8.** Expression level of *OsBAS1* in *OsTrxm*-Cas9 mutant.

Supplement: Supplementary file 8 — Figure S8 Expression level of OsBAS1 in OsTrxm‐Cas9 mutant. [file PBI-19-1511-s002.docx]
