# Supplementary material for: Repressed OsMESL expression triggers reactive oxygen species‐mediated broad‐spectrum disease resistance in rice
Source: Plant Biotechnol J. 2021 Apr 6;19(8):1511–22. doi: 10.1111/pbi.13566 (PMC8384603; doi:10.1111/pbi.13566)
Supplement: Supplementary file 2 — Figure S2 Subcellular localization of OsMESL protein. [file PBI-19-1511-s004.docx]

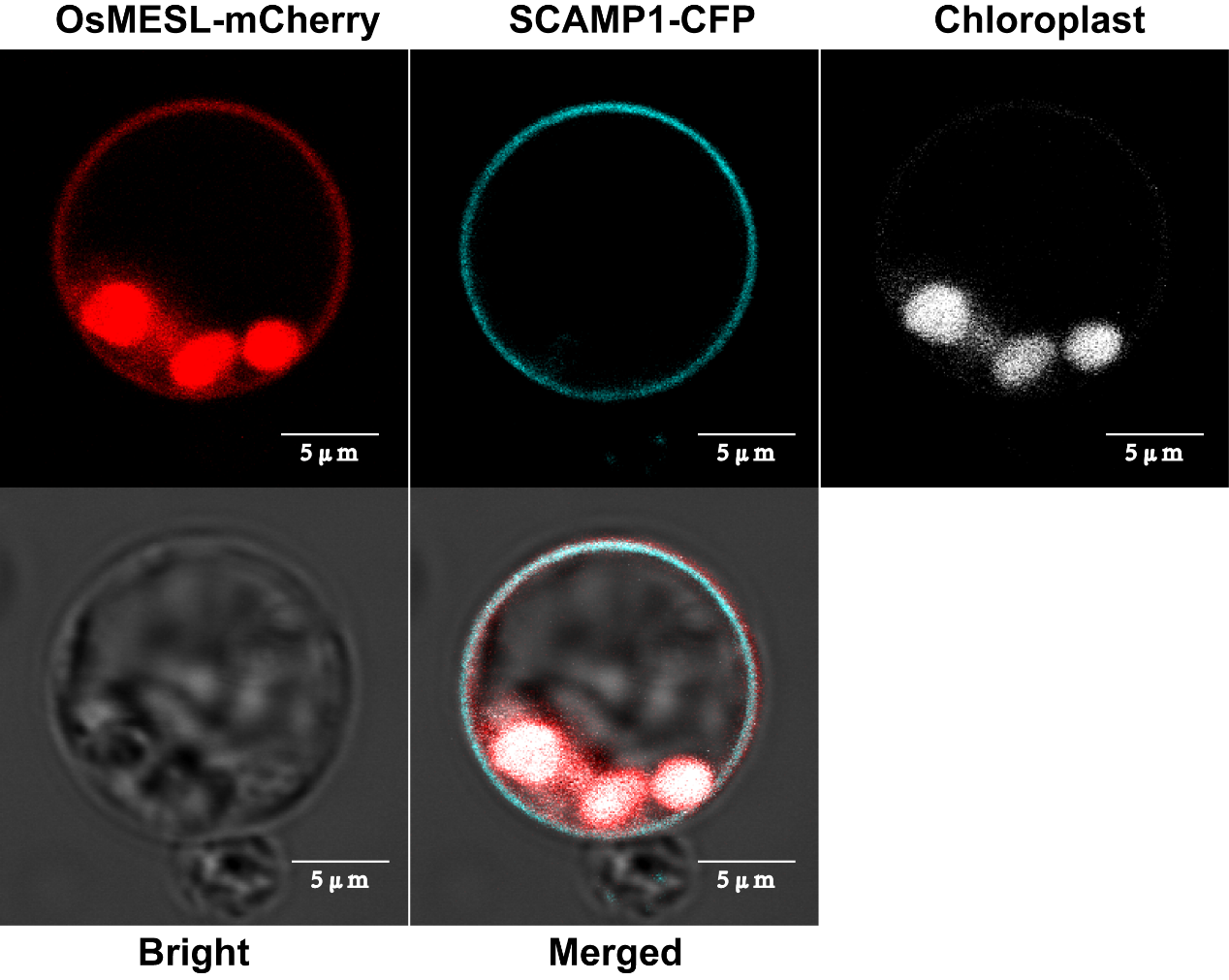


**Supplemental Figure S2.** Subcellular localization of *Os*MESL protein. *Os*MESL is localized in the chloroplast and cell membrane. *Os*MESL-mCherry, *Os*MESL fused to mCherry; ScAMP1-CFP, cell membrane marker; chloroplast, chloroplast autofluorescence. Scale bars: 5 µm.
