# Supplementary material for: Repressed OsMESL expression triggers reactive oxygen species‐mediated broad‐spectrum disease resistance in rice
Source: Plant Biotechnol J. 2021 Apr 6;19(8):1511–22. doi: 10.1111/pbi.13566 (PMC8384603; doi:10.1111/pbi.13566)
Supplement: Supplementary file 3 — Figure S3 Detectiion of copy numbers in OsMESL‐RNAi lines, OsMESL‐OE lines and osmeslcomplementation lines [file PBI-19-1511-s010.docx]

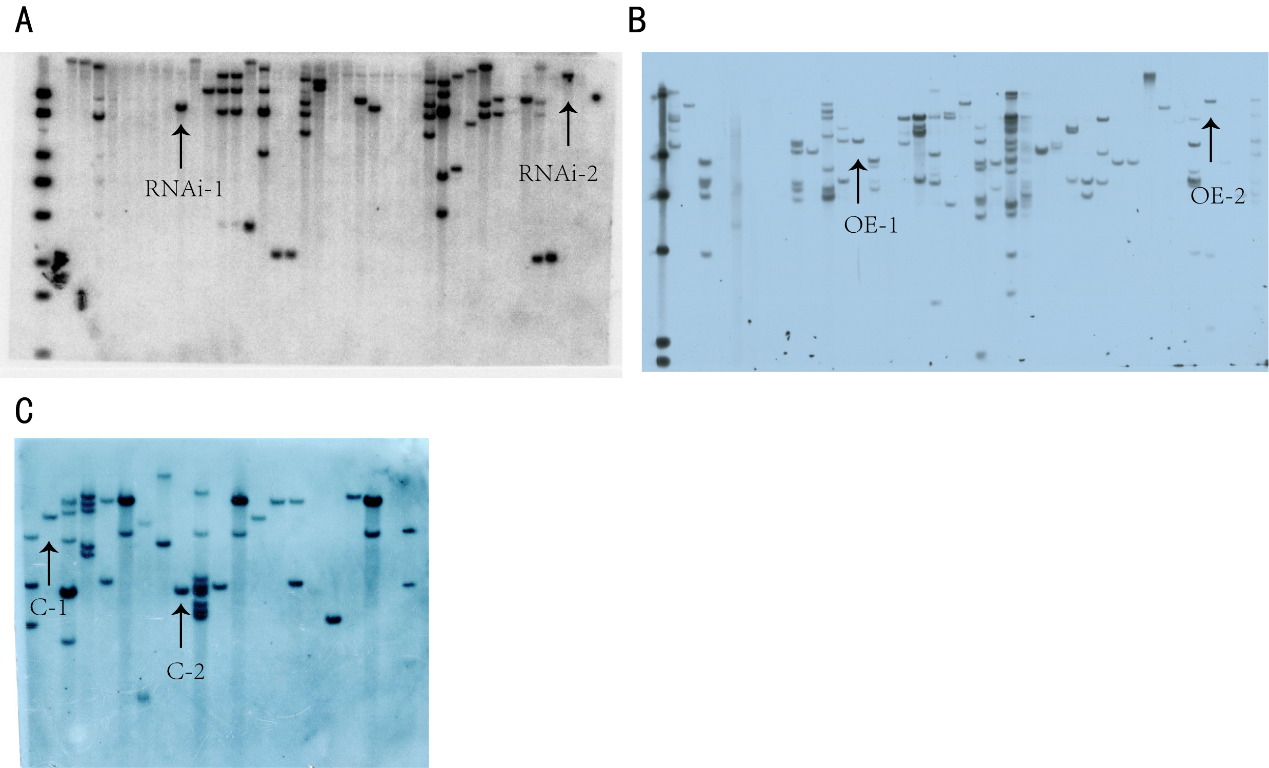


**Supplemental Figure S2.** Detectiion of copy numbers in *OsMESL*-RNAi lines, *OsMESL*-OE lines and *osmesl* complementation lines.

(**A**) copy numbers of RNAi lines, (**B**) copy numbers of OE lines, (**C**) copy numbers of complementation lines.
