# Supplementary material for: Repressed OsMESL expression triggers reactive oxygen species‐mediated broad‐spectrum disease resistance in rice
Source: Plant Biotechnol J. 2021 Apr 6;19(8):1511–22. doi: 10.1111/pbi.13566 (PMC8384603; doi:10.1111/pbi.13566)
Supplement: Supplementary file 4 — Figure S4 Phenotypes of OsTrxm OE lines inoculated with Xoo and R. solani. [file PBI-19-1511-s003.docx]

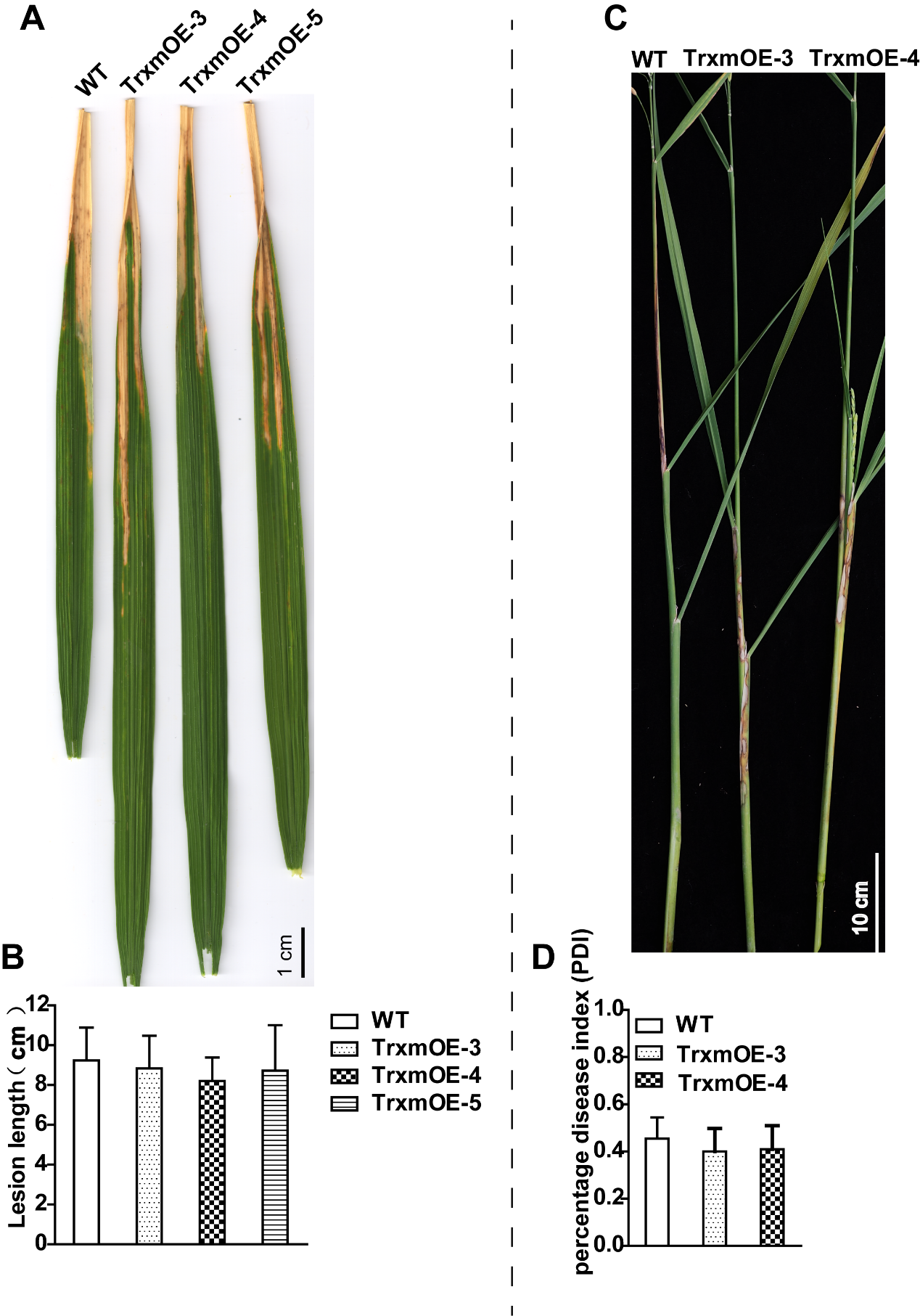


**Supplemental Figure S4.** Phenotypes of *OsTrxm* OE lines inoculated with *Xoo* and *R. solani.*

(**A-B**), *OsTrxm* OE lines showed no difference compare with WT after inoculating with *Xoo*. Values are means ± SD (n = 13).

(**C-D**), *OsTrxm* OE lines showed no resistance to sheath blight. Values are means ± SD (n = 10).
