# Supplementary material for: Repressed OsMESL expression triggers reactive oxygen species‐mediated broad‐spectrum disease resistance in rice
Source: Plant Biotechnol J. 2021 Apr 6;19(8):1511–22. doi: 10.1111/pbi.13566 (PMC8384603; doi:10.1111/pbi.13566)
Supplement: Supplementary file 5 — Figure S5 GO analysis of DEGs in osmesl and WT. [file PBI-19-1511-s001.docx]

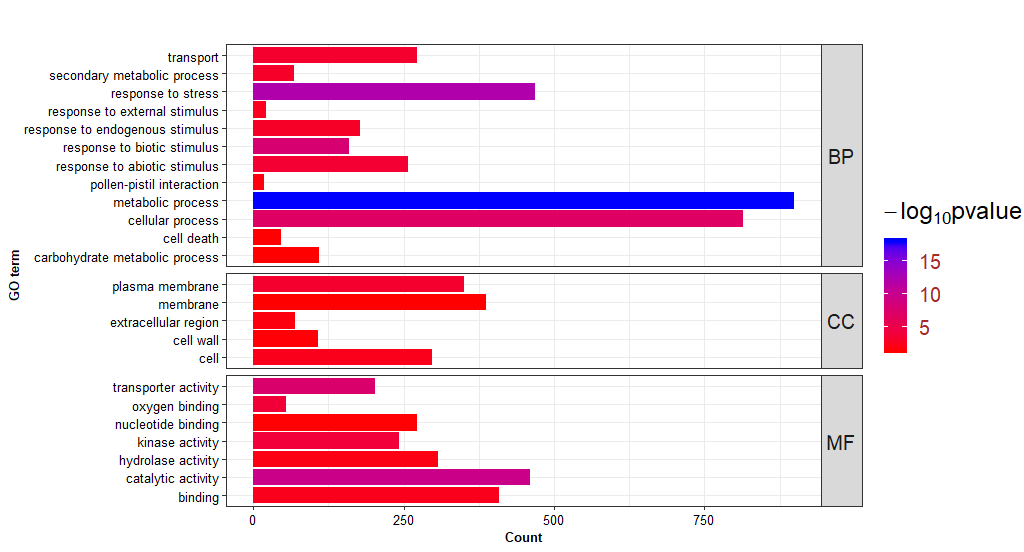


**Supplemental Figure S5.** GO analysis of DEGs in *osmesl* and WT.

BP, Biological Process, CC, Cellular Component, MF, Molecular Function.
