# Supplementary material for: Repressed OsMESL expression triggers reactive oxygen species‐mediated broad‐spectrum disease resistance in rice
Source: Plant Biotechnol J. 2021 Apr 6;19(8):1511–22. doi: 10.1111/pbi.13566 (PMC8384603; doi:10.1111/pbi.13566)
Supplement: Supplementary file 6 — Figure S6 Detection of disease resistance‐related indicators. [file PBI-19-1511-s006.docx]

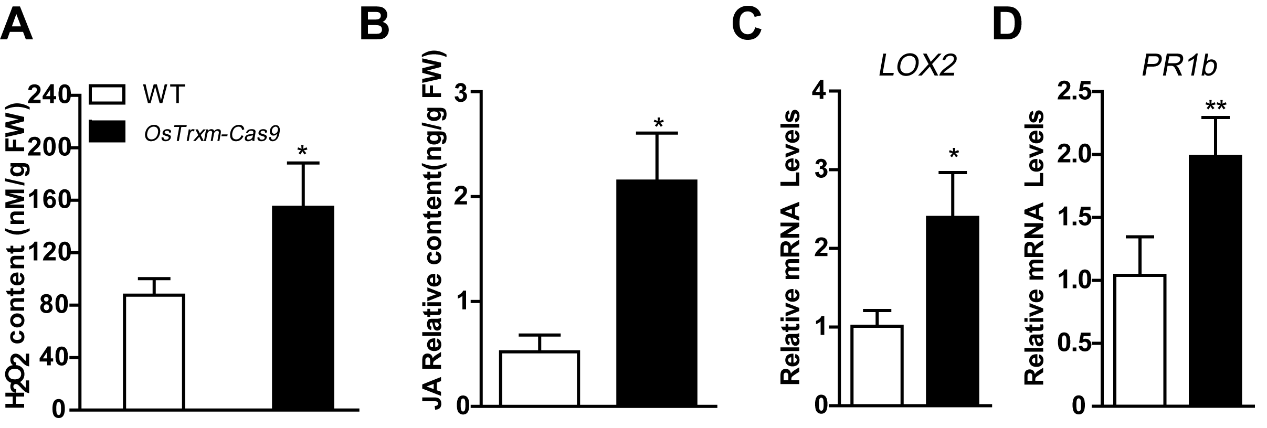


**Supplemental Figure S6.** Detection of disease resistance related indicators.

(**A**) H_2_O_2_ content in *OsTrxm*-Cas9 mutant.

(**B**) JA content in *OsTrxm*-Cas9 mutant.

(**C**) LOX2 expression level in *OsTrxm*-Cas9 mutant.

(**D**) PR1b expression level in *OsTrxm*-Cas9 mutant. (*P ≤ 0.05, **P ≤ 0.01, Student’s *t*-test).
