# Supplementary material for: Repressed OsMESL expression triggers reactive oxygen species‐mediated broad‐spectrum disease resistance in rice
Source: Plant Biotechnol J. 2021 Apr 6;19(8):1511–22. doi: 10.1111/pbi.13566 (PMC8384603; doi:10.1111/pbi.13566)
Supplement: Supplementary file 7 — Figure S7 osmesl and RNAi lines showed resistance to rice blast. [file PBI-19-1511-s007.docx]

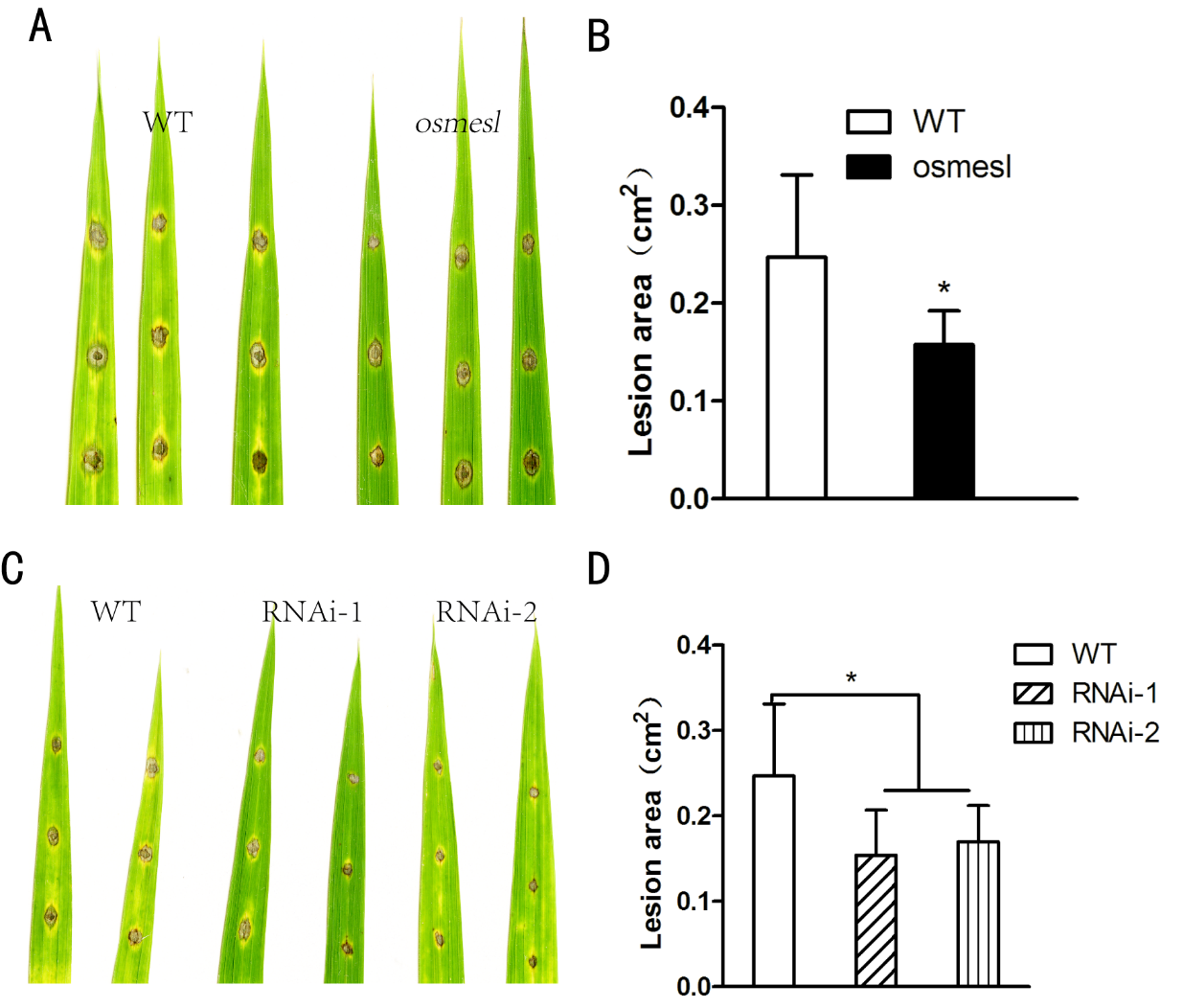


**Supplemental Figure S7.** *osmesl* and RNAi lines showed resistance to rice blast. RNAi and *osmesl* used the same WT control.

(**A**) *osmesl* showed resistance to rice blast.

(**B**) Statistics of lesion area.

(**C**) RNAi lines showed resistance to rice blast.

(**D**) Statistics of lesion area. (*P ≤ 0.05, Student’s *t*-test).
