# Supplementary material for: Repressed OsMESL expression triggers reactive oxygen species‐mediated broad‐spectrum disease resistance in rice
Source: Plant Biotechnol J. 2021 Apr 6;19(8):1511–22. doi: 10.1111/pbi.13566 (PMC8384603; doi:10.1111/pbi.13566)
Supplement: Supplementary file 9 — Table S1 Primers used in this study. [file PBI-19-1511-s009.docx]

| MESLOE-F  **Table1.** List of primers used in this study. | GGTACCGGACTCCGACGAGCAGAAGC |
| --- | --- |
| MESLOE-R | GGATCCCTTGGCTCAAGCATTACACC |
| MESLcrisprR-F | ggcAATCTCGTCGTTGGGCGTAC |
| MESLcrisprR-R | aaacGTACGCCCAACGACGAGAT |
| MESLcrisprL-F | gccGATGTCCTGCATCTCCGGGC |
| MESLcrisprL-R | aaacGCCCGGAGATGCAGGACAT |
| MESLBiFC-F | GGATCCGGACTCCGACGAGCAGAAGC |
| MESLBiFC-R | GGTACCTCATGATGCCTTGACTGGTGG |
| MESLnLUC-F | GGATCCATGGGGTCGCTGATGTCC |
| MESLnLUC-R | TCTAGATGATGCCTTGACTGGTGG |
| TrxmcLUC-F | GGATCCATGGCGTTGGAGACGTGC |
| TrxmcLUC-R | TCTAGATCAGCTGCTGACGTACTTG |
| TrxmOE-F | GAGCTC ATGGCGTTGGAGACGTGC |
| TrxmOE-R | TCTAGATCAGCTGCTGACGTACTTG |
| Trxmbifc-F | GGATCCATGGCGTTGGAGACGTGC |
| Trxmbifc-R | GGTACCGCTGCTGACGTACTTGTCG |
| TrxmcrU3-F | ggcACCATCGTATGTGTGCGTGT |
| TrxmcrU3-R | aaacACACGCACACATACGATGG |
| TrxmcrU6a-F | gccGCAGCAGCCAGTCCAAGCCA |
| TrxmcrU6a-R | aaacTGGCTTGGACTGGCTGCTG |
| QRT-Actin-F | TGGCATCTCTCAGCACATTCC |
| QRT-Actin-R | TGCACAATGGATGGGTCAGA |
| catAqrt-F | CAACCGCAACGTCGACAACTTCTT |
| catAqrt-R | TTCACCGGCAGCATCAGGTAGTTT |
| SODA1qrt-F | ATCTGGATGGGTGTGGCTAGCTTT |
| SODA1qrt-R | AGTACGCATGCTCCCAGACATCAA |
| SODBqrt-F | TCCGCCGTATAAACTTGATGCCCT |
| SODBqrt-R | TGGGTTGCCGTTGTTGTATGCTTC |
| CatBqrt-F | GCTTGCTTTCTGCCCAGCGATAAT |
| CatBqrt-R | AAATAGTTTGGGCCAAGACGGTGC |
| POD1qrt-F | ACGTCGGGGTCGCCAACAAC |
| POD1qrt-R | CGAACTCGTCCACCGACGCC |
| MESLqRT-F | CCGAGAAGGTTCTTCGGTTGA |
| MESLqRT-R | ACTGGTGGCATGGTTGCTAT |
| LOX2qrt-F | GCATCCCCAACAGCACATC |
| LOX2qrt-R | AATAAAGATTTGGGAGTGACATATTGG |
| AOCqrt-F | CTGCGATGAGCCGATGAAC |
| AOCqrt-R | TCGTTGTAGTTGCGGATCACA |
| JMT1qrt-F | CACGGTCAGTCCAAAGATGA |
| JMT1qrt-R | CTCAACCGTTTTGGCAAACT |
| PR1aqrt-F | CGTCTTCATCACCTGCAACTACTC |
| PR1aqrt-R | CATGCATAAACACGTAGCATAGCA |
| PR1bqrt-F | GGCAACTTCGTCGGACAGA |
| PR1bqrt-R | CCGTGGACCTGTTTACATTTTCA |
| PR10qrt-F | CCCTGCCGAATACGCCTAA |
| PR10qrt-R | CTCAAACGCCACGAGAATTTG |
| WRKY45qrt-F | AATTCGGTGGTCGTCAAGAA |
| WRKY45qrt-R | AAGTAGGCCTTTGGGTGCTT |
