# Supplementary material for: Repressed OsMESL expression triggers reactive oxygen species‐mediated broad‐spectrum disease resistance in rice
Source: Plant Biotechnol J. 2021 Apr 6;19(8):1511–22. doi: 10.1111/pbi.13566 (PMC8384603; doi:10.1111/pbi.13566)
Supplement: Supplementary file 10 — Table S2 Differentially expressed of defence response‐ and ROS scavenging‐related genes in osmesl plants. [file PBI-19-1511-s008.docx]

| Table S2. Differentially expressed of defense response- and ROS scavenging-related genes in osmesl plants. | | | | |
| --- | --- | --- | --- | --- |
| Locus | Annotation | | Fold change | |
| Up-regulated |  | | Log2 radio | |
| LOC_Os01g61080 (*OsWRKY24*)  LOC_Os01g40260 (*OsWRKY77*)  LOC_Os06g17900 (*Pi9*)  LOC_Os05g50890 (*OsGH3.5*)  LOC_Os06g11290 (*OsOPR1*)  LOC_Os08g35740 (*OsOPR7*)  LOC_Os02g10120 (*OsLOX1*)  LOC_Os03g08220 (*OsLOX2*)  LOC_Os03g49380 (*OsLOX5*)  LOC_Os04g37430 (*OsLOX6*)  LOC_Os08g39850 (*OsLOX8*)  LOC_Os08g39840 (*OsLOX9*)  LOC_Os07g03710 (*OsPR1a*)  LOC_Os03g18850 (*JIOsPR10*)  LOC_Os12g36830 (*RSOsPR10*)  LOC_Os01g47900  LOC_Os09g31506  LOC_Os10g13700  LOC_Os02g33540  LOC_Os04g48370  LOC_Os05g30350  LOC_Os11g11000  LOC_Os08g43680  LOC_Os09g10340  LOC_Os04g25440  LOC_Os03g30950  LOC_Os12g32760  LOC_Os11g32940  LOC_Os06g37150  LOC_Os02g04170  LOC_Os05g04550  LOC_Os08g29730  LOC_Os01g51290  LOC_Os04g29930  LOC_Os02g50240  LOC_Os07g48020  LOC_Os07g06800  LOC_Os07g03920  LOC_Os03g40100  LOC_Os06g50510  LOC_Os11g07020  LOC_Os02g35329  LOC_Os06g21380  LOC_Os08g33082  LOC_Os11g20384  LOC_Os12g01080  LOC_Os07g05380  LOC_Os04g41970  LOC_Os08g10400  LOC_Os02g49680  LOC_Os02g13870  LOC_Os09g12660  LOC_Os12g10580  LOC_Os04g47520  LOC_Os12g36940  LOC_Os01g50050  LOC_Os01g34850  LOC_Os08g29570  LOC_Os11g07020  LOC_Os08g06100 | | WRKY transcription factors  WRKY transcription factors  leucine-rich repeat protein  JA-amino acid synthetase  12-oxo-phytodienoic acid reductase  12-oxo-phytodienoic acid reductase  Lipoxygenase  Lipoxygenase  Lipoxygenase  Lipoxygenase  Lipoxygenase  Lipoxygenase  Pathogenesis related pretein  Pathogenesis related pretein  Pathogenesis related pretein  avr9/Cf-9 rapidly elicited protein  flavonol synthase  glycine-rich cell wall protein  jasmonate-induced protein  jasmonate-induced protein  heavy metal-associated protein  heavy metal transport/detoxification  CSLF3-cellulose synthase-like family  CSLF3-cellulose synthase-like family  disease resistance protein  disease resistance protein  disease resistance protein  disease resistance protein  disease resistance protein  disease resistance protein  disease resistance protein  disease resistance protein  disease resistance protein  disease resistance protein  disease resistance protein  disease resistance protein  disease resistance protein  disease resistance protein  disease resistance protein  disease resistance protein  disease resistance protein  disease resistance protein  disease resistance protein  disease resistance protein  disease resistance protein  disease resistance protein  disease resistance protein  disease resistance protein  disease resistance protein  disease resistance protein  disease resistance protein  Defensin and Defensin-like DEFL family  Defensin and Defensin-like DEFL family  Defensin and Defensin-like DEFL family  Defensin and Defensin-like DEFL family  Defensin and Defensin-like DEFL family  Defensin and Defensin-like DEFL family  disease resistance protein  disease resistance protein  disease resistance protein | | 1.37  2.52  4.18  0.87  0.39  0.31  1.47  0.23  1.12  0.43  1.33  1.95  1.39  1.90  0.50  1.38  1.40  1.30  1.61  1.05  3.20  1.50  1.18  3.63  3.27  2.06  4.03  1.43  2.54  1.44  1.74  1.10  1.84  1.86  1.14  2.42  1.13  11.55  2.77  1.26  1.43  1.18  1.21  1.21  4.76  1.52  1.26  1.67  1.11  1.01  1.75  1.34  1.15  1.26  1.54  1.02  1.29  1.86  1.43  2.17 |
| Down-regulated | | | | |
| LOC_Os09g25060 (*OsWRKY76*)  LOC_Os08g02070 (*OsMADS26*)  LOC_Os07g34570 (*OsDR8*)  LOC_Os04g51150 (*OsAOX1a*)  LOC_Os07g02440 (*OsPOD1*)  LOC_Os06g51150 (*OsCATB*)  LOC_Os05g02070 (*OsMT2b*)  LOC_Os03g17690 (*OsAPx1*)  LOC_Os04g14680 (*OsAPx3*)  LOC_Os02g34810 (*OsAPx8*)  LOC_Os02g33450(*BAS1*)  LOC_Os02g02000 (*OsHPL3*)  LOC_Os01g05585  LOC_Os04g30770  LOC_Os03g01880  LOC_Os05g38040  LOC_Os05g36270  LOC_Os03g55230  LOC_Os01g55110  LOC_Os06g15170  LOC_Os07g49310  LOC_Os01g51210  LOC_Os02g18540  LOC_Os05g34240  LOC_Os04g28250  LOC_Os06g14324  LOC_Os02g08230  LOC_Os05g35200  LOC_Os07g46670  LOC_Os05g43040  LOC_Os01g22520  LOC_Os06g02400  LOC_Os12g05370  LOC_Os05g25850  LOC_Os01g16152  LOC_Os01g48420  LOC_Os02g09940  LOC_Os02g33450  LOC_Os06g09610  LOC_Os06g42000 | | WRKY transcription factors  MADS transcription factors  disease resistance-responsive gene  alternative oxidase  peroxidase  catalase  metallothionein  ascorbate peroxidase  ascorbate peroxidase  ascorbate peroxidase  2-Cys peroxiredoxin  hydroperoxide lyase  metallothionein  catalase domain containing protein  glutaredoxin  FAD-linked oxidoreductase  FAD-linked oxidoreductase  FAD-linked oxidoreductase  glutathione S-transferase  glutathione S-transferase  glutathione S-transferase  glutathione S-transferase  glutathione S-transferase  glutathione S-transferase  glutathione S-transferase  glutathione S-transferase  glutathione S-transferase  glutathione S-transferase  glutathione S-transferase  glutathione S-transferase  glutathione S-transferase  glutathione S-transferase  glutathione S-transferase  superoxide dismutase  peroxiredoxin  peroxiredoxin  peroxiredoxin  peroxiredoxin  peroxiredoxin  peroxiredoxin | | -1.22  -3.09  -1.90  -1.00  -1.75  -1.08  -3.43  -0.41  -3.24  -0.61  -0.70  -1.31  -3.41  -1.52  -2.25  -2.07  -1.28  -7.99  -3.02  -1.96  -2.82  -2.24  -2.39  -3.05  -4.01  -1.68  -1.57  -1.31  -1.05  -1.01  -1.05  -1.06  -1.10  -0.53  -0.49  -0.21  -0.52  -0.69  -0.56  -0.45 |
